# Potential analgesic function of the clitoris in pregnant women: A feasibility study

**DOI:** 10.1371/journal.pone.0333112

**Published:** 2025-12-09

**Authors:** MM Manon Bestaux-Brethez, André Gillibert, Thibaut Sabatier, Eric Verspyck

**Affiliations:** 1 Gynaecology Obstetric Department, Rouen University Hospital, Rouen, France; 2 Biostatistics and Methodology Unit, Rouen University Hospital, Rouen, France; 3 Epidemiology and Health Promotion Department, Rouen University Hospital, Rouen, France; Universita Campus Bio-Medico di Roma, ITALY

## Abstract

Currently, the clitoris is considered to exist solely for sexual pleasure. This prospective, single-centre pilot study focuses on and evaluates an alternative function of the clitoris; pain relief during pregnancy and childbirth. Conducted between 2020 and 2023 at Rouen University Hospital, France, this feasibility study challenges the assertion of the singular function of the clitoris. The primary aim was to assess the acceptability of an intervention focusing on the potential analgesic function of the clitoris. The secondary aim was to assess pain relief. Pregnant women were offered the option of self-external massage with a vibrating device (OVD) in the clitoral suspensory ligament area for pain relief. Data were collected via a self-schedule for precise use of the method, including pain assessment before and after the use of the OVD via a self-analytic visual scale. Acceptability was considered to have been achieved if the woman used the OVD at least twice. Comparisons before and after were performed by means of a student’s t-test. With respect to acceptability, among the 32 women included, 26 (81.25%) used an OVD at least twice. Despite concerns about sexual intimacy and the sensitive period of pregnancy, this preliminary feasibility study confirmed the suitability of this mechanical vibration for the majority of the women included. In terms of pain relief, these 26 women used the OVD to manage 304 painful episodes and the feeling of relief was reported in 86.2% of these episodes. Based on a small sample size, the numerical results are obviously highly relative to the disparity of situations and feelings. However, these few consistent reports of a positive analgesic effect encourage further studies into the potential analgesic function of the clitoris.

## Context

Today, the accepted function of the clitoris is as the organ of female sexual pleasure, with the glans being an important stimulator. Anatomical knowledge of the organ covers it in its entirety (glans, pillars, bulbs and suspensory ligament). In 1998 [1] and in 2005 [2], Helen O’Connell et al., then also Lindsey A. Jackson et al. in 2019 [3] demonstrated its proximity to the urethra and the vagina and its approximate ten-centimetre dimension. The clitoris is perfectly linked to the female reproductive apparatus.

Vincenzo Puppo in 2013 [4] and Rachel N Pauls in 2015 [5], highlight the important role of the clitoris throughout the female sexual response, from arousal to orgasm. But is the function of the clitoris limited to pleasure alone?

The female orgasm (unlike the male orgasm) is not essential for the survival of the species. During reproductive intercourse, the internal part of the clitoris is stimulated by pressure exerted by the penis on the walls of the vagina [6], but what precise role does the clitoris play in the chain of actions and sensations of the act? If the clitoris only served to occasionally trigger a non-essential orgasm for procreative purposes, why did it not disappear with evolution?

Childbirth is another moment in sexual reproduction. There are reports of masturbation during childbirth [7]. Given its unique position, the clitoris cannot be indifferent to the muscular contractions of the perineum. But does it itself participate in childbirth?

In the treatment of sexual problems such as dyspareunia or vaginismus through clitoral stimulation before and during sexual intercourse, the use of accessories is recommended, particularly to reduce pain. This was shown in a study published in 2012 [8] involving more than 2,500 adult women in long-term monogamous relationships. Anatomically located at the entrance to the female reproductive system, does the clitoris guard this passage?

These questions led to the hypothesis that the clitoris has an analgesic function.

As early as 1985 in a small series of cases, and in 1988, Beverly Whipple and Barry R. Komisaruk reported that genital stimulation in women increased pain detection and tolerance thresholds, thereby inducing analgesia [9,10]. In 2023, Barry Komisaruk et al. showed that the mechanisms for obtaining pleasure and the pain circuits follow parallel, intersecting, or even common pathways [11].

How is the clitoris involved in the different stages of a woman’s life, her pain, and her pleasure? Does it act as a ‘pain controller’?

## Introduction

Epidural anaesthesia has alleviated fears of painful childbirth, but during pregnancy and the postpartum period, many clinical abdominal or perineal symptoms are painful [12]. To promote the health and welfare of mother and child, non-pharmaceutical treatment is strongly recommended during these periods. Do pregnant women have a latent, personal pain-relieving capacity at their disposal at these times? If there is a potential analgesic effect through clitoral stimulation, are they willing, given their particular situation, to pursue this personal quest and measure its effects?

It is important not to overlook the ethical and psychological aspects, the weight of taboos and the need to respect privacy, given that this involves intrusion into a part of the female anatomy that is particularly sensitive to sensations and emotions, ideas and interpretations. To enable the Committees for the Protection of Persons to authorize and oversee the study, rigorous ethical and scientific information was requested. Strict ethical guidelines were implemented to reassure women who had concerns about the safety of the proposed method for them and their babies.

Not all women masturbate and, to avoid any exclusion or statistical bias, no questions were asked on this intimate subject during the pre-inclusion visit. They were not encouraged to masturbate or pleasure themselves by experiencing a complete personal sequence from arousal to orgasm. The analgesic effect was sought through the simplest possible clitoral stimulation, explained as such, outside of a sexual context, without sensuality or sexual intercourse. No material conditions for a soothing or relaxing environment were imposed, nor were constraints on atmosphere or sensations imposed. The orgasm itself was noted as a risk that could surprise a woman who had never experienced one before.

This prospective, single-centre feasibility pilot study was conducted between 2020 and 2023 in the gynaecology-obstetrics department of the Rouen University Hospital Centre in France on a small pilot sample of 32 women.

**Fig 1:**
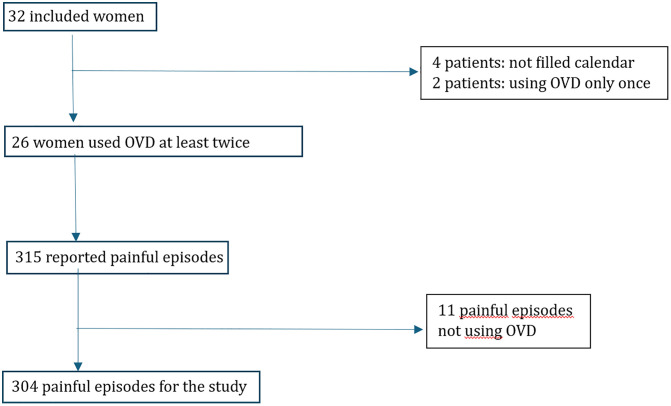
Flow-chart acceptability.

The study was classified as research on human subjects (French category 2) and approved on 14.12.2019 by the Ile de France Protection Person Committee; however, the study was prolonged to 02.08.2021 due to the coronavirus disease 2019 (COVID-19) pandemic and registered at ClinicalTrials.gov. (Fig 1).

## Method

This is massage and not masturbation. In this study, using a mechanical vibrator was deemed more effective, as the vibration is expected to last longer than other methods of clitoral massage.

Clitoral stimulation was offered without any vaginal penetration. It seems possible to reach the ischiopubic and bulbospongiosus muscles surrounding the clitoris through external vibration applied on the pubis area. This causes perineal relaxation that is beneficial to the patient. Thus, it did not seem necessary to propose internal stimulation, which could be effective, but possibly less acceptable to all pregnant women.

The clitoral glans, which is too sensitive and too innervated, and which can sometimes trigger rapid orgasm, was not selected. Women were not encouraged to produce a clitoral erection through direct intervention on the glans. While it is likely that vibration will induce blood flow throughout the clitoris and nervous responses closely linked to pleasure, it should also be remembered that not all women are accustomed to this path to sexual pleasure. To achieve the desired balance between pain and pleasure, it did not seem appropriate to aim for the peak of pleasure (orgasm) as quickly as possible, but rather to remain in the plateau phase of the human sexual response described by Masters and Johnson [13].

**Fig 2:**
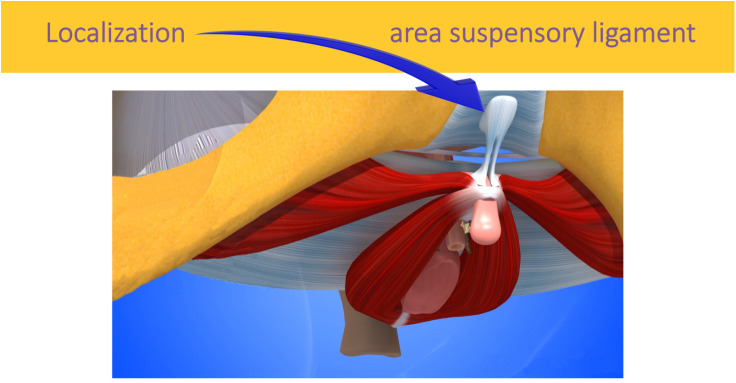
Device positioning area.

To optimize mechanical vibration and maintain a relaxation sequence, the suspensory ligament of the clitoris and its attachment to the pubic symphysis has been proposed as the focal point. (Fig 2) Even through fabric (and therefore without the need to undress), vibration applied to a hard part, the pubic bone, is transmitted and transformed (by resonance, phases, and phase oppositions) throughout the chain formed by the fasciae [14], nerves, muscles, and the clitoris itself.

## Material

It was necessary to choose a device that was suitable for the method, identical for all women included, and as harmless and non-aggressive as possible.

Phallic and penetrating sex toys were ruled out from the outset, as were pneumatic suction devices for the clitoris alone.

**Fig 3:**
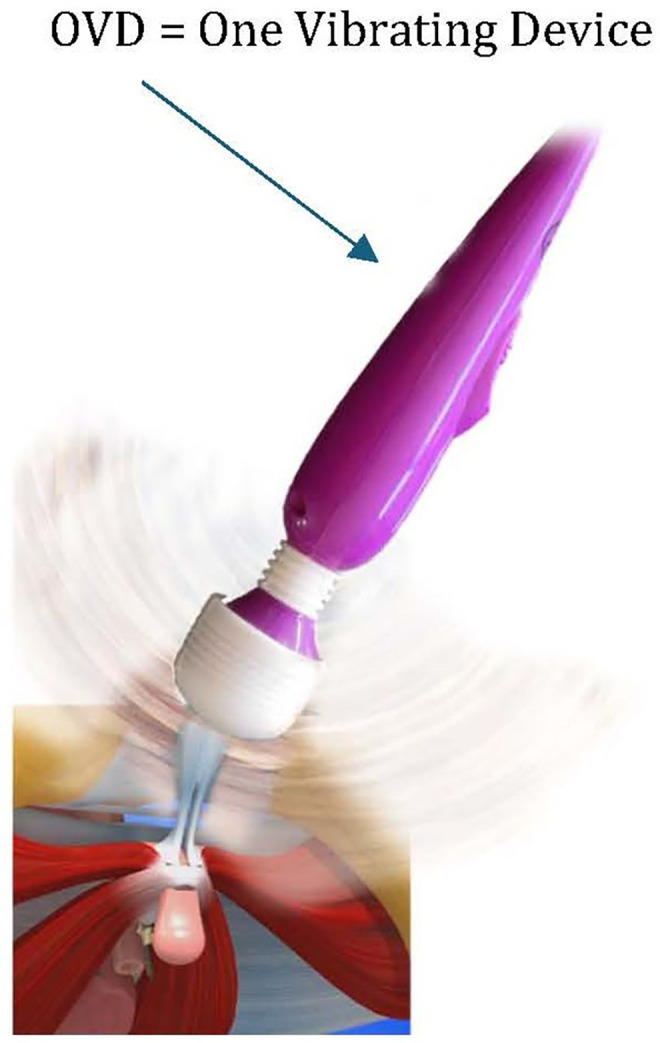
The OVD (One Vibrating Device).

The choice fell on a very simple vibrator with a CE electrical safety certification (Fig 3). The vibration produced is a mechanical vibration obtained by the imbalance of a moving mass of a small electric motor. The frequency variation of the vibratory signal depends on the speed of rotation (adjusted by a simple rotary potentiometer), thus providing a vibration range of several tens of hertz, with successive frequencies enriched by several harmonics.

Once the One Vibrating Device (O.V.D.) is placed on the body, either directly or through fabric, the vibration obtained is highly sensitive to interaction with the chosen surface. The treatment is adjusted by the patient according to how she feels. The flexible connection (spring) between the motor body and the vibrating head attenuates high frequencies and prevents the woman from experiencing excessive irritating surface sensations. The OVD has low torque. This has the advantage of keeping the vibration constant and soothing, as any effort by the pregnant woman to press the OVD against herself is offset by a slowing down of the device. By adjusting the switche knob, she can find the right setting for a vibration that is neither unpleasant nor too strong.

The device used allows the pregnant woman to physiological remain in tune with her body while maintaining a certain psychological distancing. This is a welcome safeguard at a time when the two issues of motherhood and sexuality are not easily reconciled.

### Inclusion criteria

When the word clitoris is mentioned, the conversation is often interpreted as sexual and therefore delicate, intimate or even off-limits. To avoid any preconceptions about the research and any bias in understanding, the inclusion criteria were kept as unrestrictive and unbiased as possible.

The poster and flyer distributed in the obstetrics department only mentioned pain research without providing any further details. The words “clitoris” and “stimulation” were mentioned only during the pre-inclusion visit in reading material discussions about the information, and the consent document to be signed by the woman (N.I.C.E).

During the pre-inclusion visit, two women insisted on disclosing that they had been subjected to female genital mutilation and gave their consent for this personal information to be used.

A prior understanding of the protocol was requested in order to clarify the act of vibration. Viewing a video by Patrice Thiriez of the University of Lyon, France, during the pre-inclusion visit was very helpful in educating the pregnant women by showing a 3D image of the clitoris, clearly located within the perineum [15].

### Evaluation criteria

The principal outcome of acceptability is that a pregnant woman the procedure at least twice after inclusion. In the absence of previous clinical research, this low figure of two applications avoids introducing bias through random single applications.

The results regarding pain relief, details and comments were recorded in writing on a calendar. Stimulation actions could be recorded, dated and annotated simply, without the emotional or fantasized aspects of sexual intimacy becoming too involved. Pain could be rated using the Visual Analogic Scale widely used at Rouen University Hospital.

An optional visit was scheduled around the 32nd week of amenorrhea (32WA) to ensure that the schedule was understood and to answer any questions. During the COVID-19 pandemic, this visit was frequently replaced by a phone call.

### Inclusions

**Fig 4:**
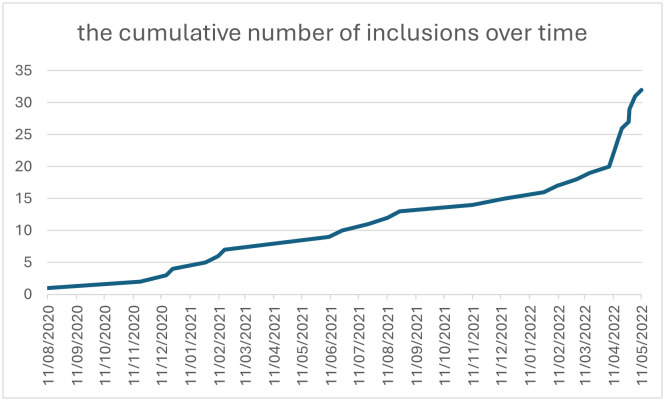
Frequency of inclusions.

With an average of 56 women giving birth each week in the obstetrics department of Rouen University Hospital, the projected rate of inclusion was estimated at 2.8 per week. From 11 August 2020–11 May 2022, it was actually lower than expected, due to the various waves of Covid. (Fig 4) The cancellation of childbirth preparation classes significantly reduced public awareness of the study and the involvement of medical staff. The initial duration of the study was therefore extended. 

#### Feasibility results.

In this context, the first 32 patients who attended the pre-enrolment visits all gave their consent. 4 of them were lost to follow-up or did not return the calendars, and 2 used the method only once. According to the predefined criterion (at least two uses of the device), the acceptance rate of 81.25% (26 women out of 32, 95% confidence interval (CI): [63.356–92.79]) indicates a good overall intention to utilise the procedure.

Compliance with the procedure did not appear to pose any difficulties for the remaining 26 patients. Of the 315 episodes reported in the calendars, only 4 were classified as ‘non-use of the procedure’ (due to contractions that were too short and physical impossibility) and 7 were due to misuse by a patient who had placed the OVD on the sacrum (rather than on the pubic symphysis) to relieve lower back pain. The procedure was therefore strictly followed in only 304 interventions.

**Fig 5:**
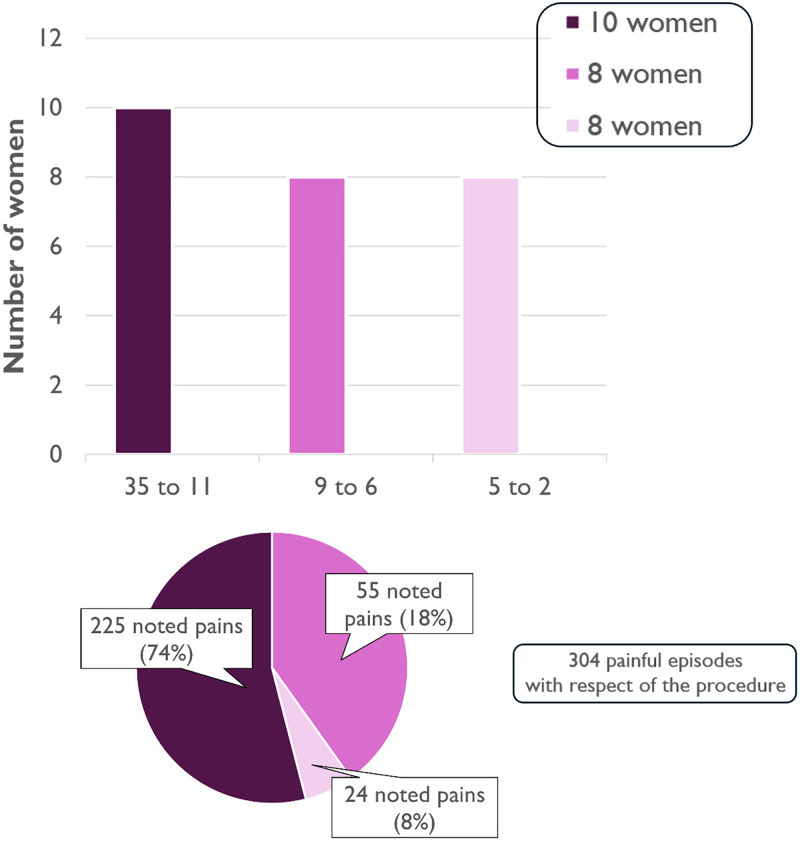
304 respected procedures.

However, given the wide variability in pain and how it is experienced, the level of intervention is very diverse and depends on each individual’s motivations. Thus, it is useful to detail groups (Fig 5).

With 35–11 uses of OVD during the inclusion period, sometimes several times a day, 10 of the 26 women performed 225 of the 304 reported painful episodes, which is 74%. They are the ones who invested the most and made extensive use of the program. They made it their own. They wrote comments in the calendars. The two women who had undergone excision were among them. (Group 1).

With 9–6 uses of OVD during the inclusion period, rarely more than once a day, 8 performed 55 (18%). This is a diverse group with a variety of reasons and motivations. (Group 2).

With 5–2 uses of OVD during the inclusion period, 8 women recorded the remaining 24 uses (8%). This represents cautious and limited acceptability. (Group 3).

### Feasibility in the presence of another person

This apparent feasibility of the procedure also has a significant limitation. Application of the procedure in the presence of another person has often been lower and was stopped in the final phase of labour, proof of the intimate nature of the requested act. Nevertheless, the only two exceptions were patient 7 with four uses in the presence of medical staff for vaginal examinations and patient 18 in the delivery room, who interrupted application due to discomfort with the monitoring device.

### Relief

**Fig 6:**
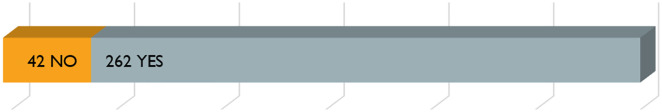
Binary relief box.

The secondary data collected for this feasibility study was the answer YES or NO in the box labelled ‘relief’. The answer YES on the calendar was checked 262 times, representing 86.18% of device applications. (Fig 6). An overall positive effect of relief was observed.

### VAS scores

**Fig 7:**
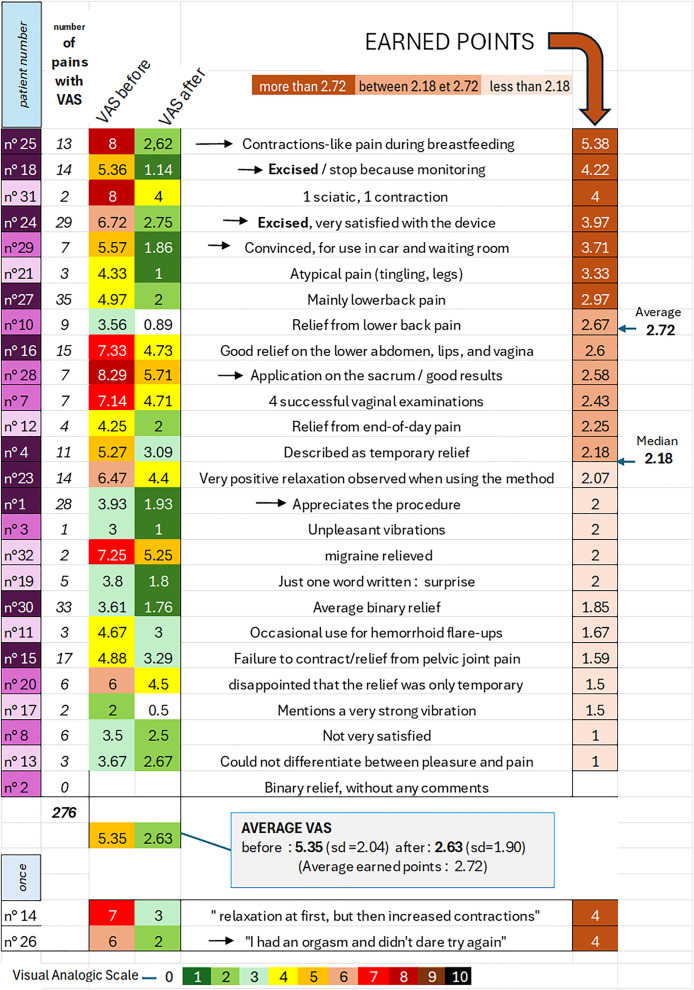
Reported pains with VAS.

The following data did not claim to establish reliable measures of the analgesic efficacy of the device itself, but rather to provide an initial assessment of the observed effect. It had been indicated that information on the VAS (Visual Analog Scale) was optional. Nevertheless, these VAS data were recorded for 276 painful episodes. When recorded before and after use, the average VAS score before use was 5.35 (sd = 2.04) and after use was 2.63 (sd = 1.90), which correspond to 2.72 earned points. (Fig 7).

These figures confirm the general impression of relief. They are a sign of a positive assessment of the method, without being an objective measurement of it.

The variability of VAS scores, sample size, and written comments allow us to mention a few interesting special cases (indicated by an arrow) for better understanding of this analgesic effect in the future.

7 women achieved above-average gains in VAS scores (2.72). The best score of 5.38 (average over 13 actions) was achieved by patient 25, who experienced 10 episodes of postpartum pain related to breastfeeding. High scores were also noted for the two women who had undergone female genital mutilation, patients 18 and 24 (4.22 for 14 procedures and 3.97 for 29 procedures, respectively). Of note is patient 29, who was convinced of its effectiveness and used it successfully in the car on the way to the hospital and in the waiting room.

6 women between 2.18 (median score) and 2.72 (average) confirmed significant pain relief and a highly variable frequency of procedures. Note patient 28, who, for certain pains, placed the OVD on her sacrum.

12 women were disappointed with less than 2.18 points gained. The device was used occasionally. Application provided little difference in pleasure and only slight relief during strong contractions.

2 orgasms were reported in the comments. One was positive (patient 1), improving the pain-relief effect during sexual intercourse. The other (patient 26) was also positive in terms of relief (6/2) but psychologically blocked any further attempts (only one application of the procedure).

### Location of pain reported

On the calendars, the ‘contraction’ box was ticked 118 times. 3 women (patients. 15, 24 and 25) reported 64 contractions, confirming a wide disparity on this subject.

**Fig 8:**
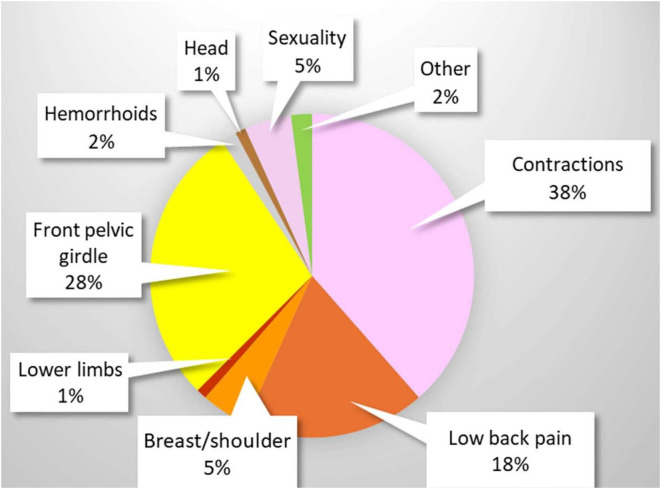
Location of pain.

Thanks to comments (192) from several women, we have an idea of where the pain is located in the body (Fig 8), bearing in mind that they sometimes reported several locations per episode. Unsurprisingly, the pelvic region is the most common location (73 uterus, 55 front pelvic girdle, 35 lower back).

## Discussion

What is the cause of the analgesic effect observed?

Does this stimulation maintain sufficient relaxation of the entire perineal area to counteract painful sensations through a gate control reaction [16]?

Or is it the quality of the mechanical vibration on the suspensory ligament of the clitoris that induces a response in the clitoris itself, perhaps through its Krause corpuscles, which are highly sensitive vibration detectors [17]?

In order to speak of an analgesic function of the clitoris, proof would be needed that the clitoris acts specifically on pain control mechanisms. Further studies could help to clarify this point by measuring endorphin and neurotransmitter levels.

Barry R. Komisaruk and Beverly Whipple showed in 2000 [18] that endogenous opioids may be just one of many neurotransmitters responsible for analgesia induced by genital stimulation.

New investigations into the physiological changes in the clitoris during this transitional phase of analgesia (e.g., erection of the corpora cavernosa, stimulation of the muscles in the area, etc.) may also provide some answers.

Further research into other female pains (dysmenorrhea, endometriosis, etc.) and rigorous study of other locations for mechanical vibrations in different areas of the perineum are also needed to extend the conclusions beyond the period of pregnancy. The individual initiative of applying vibrations to the sacrum (outside the defined protocol), the effectiveness of the method for pain related to breastfeeding (postpartum, etc.) and the particular experience of the two women who had undergone female genital mutilation raise questions on this point and suggest new areas of study.

As for the difficulty of implementing this practice in the presence of caregivers and medical staff, we are counting heavily on improvements to the vibrating device used. Making it compatible with the equipment used during childbirth and presenting it as a genuine, effective medical device without any reference to the world of sex toys are now priorities.

## Conclusion

The feeling of relief experienced when using the device with the method described is sufficient to warrant further, more comprehensive, qualitative research into this little-known analgesic capacity of the clitoris.

Female orgasm and sexuality were not the focus of this research, and the commonly held belief that the clitoris is “the only organ exclusively reserved for sexual pleasure” has not been reaffirmed. 26 out of 32 women agreed to test the method without hesitation, without fear for the child, or moral qualms.

The study clearly shows that pregnant women can actively participate in managing their pain beyond physical apprehensions and taboos. However, the degree to which they embraced the vibrating device and the method itself varied greatly. Depending on the severity or nature of their pain, some women were much more motivated than others, achieving better results through more deliberate use of this analgesic effect, sometimes surprising to them.

This diversity and inequality in the experience of pain raises questions about medical support. This study shows that offering clitoral stimulation is acceptable to women but never morally straightforward. The intimacy required for the development of such a therapeutic method can only be achieved with caution, restraint and benevolence. Much work remains to be done to raise awareness in the medical community. This publication is part of that effort.

In conclusion, this study demonstrates an analgesic effect that is easy to use at the peak of pain and useful for achieving relaxation and a degree of calm in cases of prolonged pain.

The clitoris is a dynamic organ, strongly connected to muscles, fasciae, nerves and all the female reproductive organs. Mobilizing it reduces pain. We have observed this effect particularly when women make an effort to focus on it.

This research offers new perspectives for applying this pain-relieving effect as a complement to conventional medical efforts in the broad field of combating female pain.

## Supporting information

S1 File Protocole FAC V3 without logo. (PDF)

S2 File Translation Protocole V3 without logo. (PDF)
