# Supplementary material for: Potential analgesic function of the clitoris in pregnant women: A feasibility study
Source: PLoS One. 2025 Dec 9;20(12):e0333112. doi: 10.1371/journal.pone.0333112 (PMC12688119; doi:10.1371/journal.pone.0333112)

PROTOCOLE D'ETUDE IMPLIQUANT LA PERSONNE HUMAINE
MENTIONNE AU 2° DE L'ARTICLE L.1121-1 du CSP

Recherche par la femme enceinte de la potentielle fonction analgésique du clitoris : Etude de faisabilité *F.A.C.*

2018/0415/HP

2018-A03483-52

Version 3 du 28/06/2021

Investigateur Principal :
Professeur Eric VERSPYCK,
Service de Gynécologie Obstétrique
CHU de Rouen
1 rue de Germont 76031 Rouen Cedex
Tél. : 02 32 88 82 44

Co-Investigatrice :
Dr Marie-Madeleine, Manon BESTAUX-BRETHEZ
Service de Gynécologie Obstétrique
CHU de Rouen
1 rue de Germont 76031 Rouen Cedex
Tél. : 02 32 88 82 44

Promoteur :
Délégation à la Recherche Clinique et à l'Innovation
CHU de Rouen
1 rue de Germont 76031 Rouen Cedex
Tél. : 02 32 88 82 65
Fax : 02 32 88 82 87

Méthodologiste et Biostatisticien :
Dr André GILLIBERT
Unité de Biostatistique et de méthodologie
CHU de Rouen
1 rue de Germont
76031 Rouen Cedex
Tél : 02.32.88.84.94

Synopsis

**Titre de l'étude code Promoteur**	Recherche par la femme enceinte de la potentielle fonction analgésique du clitoris (F.A.C.): Etude de faisabilité<br>2018/0415/HP
**Investigateur principal**	Professeur Eric VERSPYCK<br>Service de Gynécologie Obstétrique<br>CHU de Rouen, 1 rue de Germont 76031 Rouen cedex
**Classification**	Protocole d'étude impliquant la personne humaine mentionné au 2° de l'article L.1121-1 du CSP
**Promoteur**	Délégation à la Recherche Clinique et à l'Innovation<br>CHU de Rouen, 1 rue de Germont 76031 Rouen Cedex<br>Tél. : 02 32 88 82 65 Fax : 02 32 88 82 87
**Population concernée**	Femmes enceintes comprenant le français
**Objectifs de l'étude**	**Objectif principal** :<br>Evaluation de l'acceptabilité par la femme enceinte de la recherche de la potentielle fonction analgésique du clitoris.<br><br>**Objectifs secondaires** :<br>Evaluer le soulagement de la douleur conséquent à l'activation de la fonction analgésique du clitoris.
**Critères d'évaluation**	**Critère d'évaluation principal** :<br>Parmi les patientes ayant eu la visite de pré-inclusion, proportion de celles qui ont été incluses et ont appliqué la procédure au moins deux fois<br><br>**Critères d'évaluation secondaires** :<br>1/ Perception d'un soulagement d'une douleur chez la femme enceinte par mise en œuvre de la procédure, au moins sur deux tentatives, renseignée par le calendrier FAC (Fonction Analgésique du Clitoris).<br>2/ taux d'inclusion parmi les patientes après la visite de pré-inclusion (décomposition du critère de jugement principal).<br>3/ taux d'application de la procédure (au moins deux fois) parmi les femmes incluses (décomposition du critère de jugement principal)
**Type de l'étude**	Étude prospective, à un seul groupe (pas de groupe contrôle), mono centrique, pilote
**Critères d'inclusion**	• Femme enceinte, quelque soit le terme, primipare ou multipare<br>• Âge ≥ 18 ans<br>• Patiente ayant lu et compris la lettre d'information et signé le formulaire de consentement<br>• Patiente affiliée à la Sécurité Sociale

**Critères de non-inclusion**	• Inaptitude à comprendre le français ou l'information,<br>• Personne privée de liberté par une décision administrative ou judiciaire<br>• Personne placée sous sauvegarde de justice<br>• Personne sous-tutelle ou curatelle
**Procédure de la recherche**	En cas de douleur, massage par apposition de l'outil thérapeutique vibrant OVD (Outil Vibrant de Détente conforme CE) entre la symphyse pubienne et le genou du clitoris sur le ligament suspenseur, pour la détente des muscles ischio caverneux et bulbo spongieux, permettant l'érection des corps caverneux du clitoris. Evaluation par le calendrier FAC (Fonction Analgésique du Clitoris) renseigné par la patiente.
**Nombre théorique de patients à inclure**	32
**Nombre prévu de centres**	1
**Analyse Statistique des données**	L'analyse principale sera faite par estimation du pourcentage de femmes ayant appliqué la procédure au moins deux fois parmi celles qui ont eu la visite de pré-inclusion.<br>D'un point de vue statistique, il ne sera pas possible de conserver des informations détaillées sur les femmes venues à la visite de pré-inclusion mais non incluses ; par contre le volume de visites de pré-inclusion pourra être évalué précisément par enregistrement du nombre de visites.<br>Un pourcentage supérieur à 25% sera considéré comme acceptable.<br><br>Étant donné qu'il s'agit d'une étude de faisabilité, les seules données de la littérature étant des cas rapportés très isolés, il est préférable de réaliser l'étude sur un petit échantillon pilote. Avec 64 patientes venant à la visite d'information, 32 incluses et 16 utilisant la procédure, l'incertitude sur le taux 16/64 correspondra à un facteur 2,5 entre la borne haute et la borne basse de l'intervalle de confiance.<br>C'est-à-dire, l'intervalle de confiance attendu est (15% à 37%).
**Calendrier prévisionnel**	Durée de la période d'inclusion = 24 mois<br>Avec environ 56 femmes accouchant par semaine, en comptant 1/20 qui consultera à la visite de pré-inclusion sexuelle, soit 2,8 femmes par semaine. Avec un taux d'inclusion de 50% de ces femmes, le rythme d'inclusion sera de 1,4 par semaine. Il faudra alors 23 semaines soit environ 24 mois pour l'inclusion.<br>Durée de suivi par patiente : maximum 9 mois<br>Nombre de Visites: 3<br><br>Durée totale : 33 mois

Sommaire

Synopsis **2**
Sommaire **4**
1. Justification scientifique et description générale de l'Etude **6**
2. Objectifs de l'Etude **12**
2.1. Objectif principal 12
2.2. Objectif secondaire 12
3. Conception de l'Etude : **13**
3.1. Critères d'évaluations principaux et secondaires 13
3.2. Description du type de l'Etude 14
4. Sélection des personnes de l'Etude **14**
4.1. Critères d'inclusion 14
4.2. Critères de non-inclusion 14
4.3. Modalités de recrutement 14
5. Procédures de l'Etude **15**
5.1. Description de la procédure évaluée lors de la recherche : 15
5.2. Risques de la procédure: 16
5.3. Calendrier FAC (Fonction Analgésique du Clitoris) 16
5.4. Traitements/dispositifs/procédures associés interdits dans le cadre du protocole 17
5.5. Traitements/dispositifs/procédures associés autorisés 17
6. Déroulement de l'Etude **17**
6.1. Calendrier de l'étude 17
6.2. Schéma de la recherche APPEL 18
6.3. Tableau récapitulatif du suivi patient 18
6.4. Visite de pré-inclusion - Visite V0 19
6.5. Visite d'inclusion – V 1 19
6.6. Suivi par appel téléphonique (2 semaines après V1) 21
6.7. Suivi par appel téléphonique (début du 3ème trimestre) 21
6.8. Visite de suivi – V2 21
Visite de fin de l'Etude (dernière visite réalisée par la patiente) – V3 21
6.9. Règles d'arrêt de l'Etude 21
7. Sécurité des personnes **22**
Gestion des Evènement Indésirables 23
8. Statistiques : **23**
8.1. Description des méthodes statistiques 23
8.2. Nombre prévu de personnes à inclure dans l'Etude 24
8.3. Degré de signification prévu 24
8.4. Critères statistiques d'arrêt de l'Etude 24
8.5. Modalités de remplacement 24
8.6. Réalisation d'une analyse intermédiaire 24
9. Droit d'accès aux données et documents source. **24**
10. Contrôle et assurance qualité **26**
10.1. Gestion des données relatives à l'étude 26
10.1.1. Saisie et stockage des données 26
10.1.2. Modalités de traitement, vérification et validation des données 26
10.1.3. Destinataire des données traitées 26
10.2. Contrôle Qualité 27
10.3. Audit et inspection 28
11. Conservation et archivage des données relatives à l'Etude **28**
12. Considérations éthiques et réglementaires **29**

13. Règles relatives à la publication et à la communication.............................. 30
14. Références à la littérature scientifique.. 31
15. Liste des abréviations... 33
16. Liste des annexes : 34

1. Justification scientifique et description générale de l'Etude

La grossesse est pour la femme un phénomène physiologique pouvant s'accompagner, au cours de son évolution dans le temps, de manifestations cliniques douloureuses abdominales ou périnéales parmi lesquelles les contractions utérines « fausses » tout au long de la grossesse ou « vraies » à l'approche du terme. En première intention, la démarche de lutte contre la douleur est recommandée non médicamenteuse pendant cette période. La fonction analgésique du clitoris a sa place dans ce contexte, pour peu que la parturiente envisage d'y puiser les bénéfices analgésiques attendus, puis qu'elle veuille et puisse le faire.

En dehors de la grossesse, en consultation de sexologie, les témoignages abondent sur l'amélioration des dyspareunies et la lutte contre le vaginisme par stimulation du clitoris avant et pendant le rapport sexuel. Ils documentent de très nombreux actes sexuels plus ou moins efficaces, auto-stimulation du bouton du clitoris ou préliminaires effectués par le partenaire. L'utilisation d'accessoires dans un contexte d'éducation sexuelle à destination d'amélioration de la vie sexuelle, et notamment la réduction des douleurs, est recommandée, comme le prouve une étude en 2012 sur plus de 2500 femmes adultes dans des relations monogames de longue durée [1]. C'est la fonction analgésique du clitoris qui est recherchée. En phase d'excitation sexuelle, un état analgésique lié à l'érection des corps caverneux du clitoris permet et facilite le rapport sexuel. Endorphines et cortisol sont déversés dans l'organisme pour un effet décontractant et désinhibant [2] .

En amont de ces actes sexuels, nous émettons l'hypothèse d'une fonction analgésique liée à la fonction globale de reproduction qui donne une place plus utile au clitoris dans l'appareil génital féminin. La nature ne faisant pas plaisir par plaisir, il serait étonnant que le clitoris ne soit là pour rien.

Le clitoris n'est pas toujours représenté dans l'appareil génital. Il est même pratiquement exclu de l'appareil de reproduction féminin. La fonction communément admise du clitoris est celle d'être exclusivement réservée au plaisir sexuel féminin même si on peine à expliquer qu'une si forte minorité de femmes ne connaissent pas cette « fonction plaisir ». La connaissance de l'anatomie féminine est inégalement partagée par les femmes. L'autostimulation du clitoris est loin d'être aussi systématique que la masturbation masculine. L'orgasme féminin semble également plus difficile d'accès.

A trop vouloir parler de plaisir sexuel dans le contexte si particulier de la grossesse on affronte directement les barrières intimes et on jette souvent un trouble supplémentaire (psychologique, culturel ou social) dans une période déjà délicate. Autant se placer du côté de la connaissance et permettre à chaque patiente d'être une actrice éclairée de son propre accouchement.

Les connaissances anatomiques récentes et documentées d'Helen O'Conell [3]. Odile Buisson et Pierre Foldès [4] définissent le clitoris comme un organe à part entière dans l'appareil génital féminin. Il est enfin représenté dans sa totalité dans un manuel scolaire. Ses branches enserrent le vagin. Les muscles ischio caverneux et bulbo spongieux participent au contrôle des corps caverneux du clitoris et en permettent ou non l'érection. Il n'est plus objectivement le « petit » bouton, le « petit » pénis, la « petite gâchette ». Mais son image ne s'en améliore pas pour autant : il continue à être immédiatement associé, accolé et par analogie au masculin accompagné du tabou sexuel par excellence : la masturbation.

Le manque de données scientifiques sur les fonctions du clitoris est un problème d'histoire et d'interprétations : comment étudier un organe non décrit anatomiquement pendant des siècles et, corrélativement, comment étudier une fonction physiologique différente de l'idée imposée (organe exclusif du plaisir sexuel féminin) puisque cette interprétation unilatérale repose sur la même méconnaissance anatomique ?

Pour les femmes qui ont une connaissance éclairée de leur anatomie (par autostimulation), il est souvent relaté que la masturbation est une technique éprouvée de diminution des douleurs des règles ainsi que des douleurs lombaires pendant la grossesse.

Whipple *et al* ont prouvé en 1988, sur une petite série de cas que la stimulation génitale des femmes élevait les seuils de détection et de tolérance de la douleur [5]. Des femmes volontaires saines, de 26 à 57 ans ont été recrutées. La stimulation vaginale et clitoridienne augmentait le seuil de détection d'une douleur provoquée par compression standardisée (jusqu'à 1 kg) des doigts de la main gauche. Le faible nombre de cas est heureux eu égard à la méthode utilisée, la provocation d'une douleur ne faisant plus partie fort heureusement des méthodes éthiques acceptées aujourd'hui. A noter qu'il s'agit de masturbation (self-stimulation) et d'une confusion anatomique aujourd'hui dépassée (entre clitoris et vagin).

Un mémoire de maïeutique belge, présenté en 2018, décrit des témoignages de stimulation du clitoris aux moments douloureux de l'accouchement qui font chuter la douleur de manière

significative [6]. Ces cas rapportés ont été identifiés par une étudiante sage-femme qui les a recrutés sur un groupe Facebook prônant l'accouchement à domicile comptant environ 5000 adhérentes. Le message d'invitation précisait qu'il était recherché des femmes ayant ressenti l'envie ou le besoin de se masturber pour se soulager de la douleur pendant l'accouchement. Seules les femmes n'ayant pas eu de péridurale ont été incluses. Cinq témoignages de femmes décrivant un soulagement de la douleur des contractions utérines pendant l'accouchement ont été recueillis dans des entretiens téléphoniques qualitatifs.
Il existe manifestement un très fort biais de sélection et cela ne reste que des cas rapportés.

Notons qu'il s'agit d'une masturbation de femmes connaissant assez leur corps et dans des conditions si particulières, en volontaire rupture des soins hospitaliers, qu'il faut accorder à ces témoignages leur seule qualité de témoignages.

Aucune des femmes interrogées n'a eu ni recherché d'orgasme comme si seule la phase d'érection était à prendre en compte. Le terme d'accouchement « orgasmique » est donc inadapté pour ces cas référencés.

Pour y voir plus clair et informer correctement nos patientes, nous détaillons l'exemple de l'ocytocine, hormone contractante chez la femme enceinte, que l'on déclare partout comme l'hormone commune et le trait d'union entre la femme et la mère, l'accouchement et le plaisir.

Déjà en 1987, il a été prouvé que l'ocytocine fait partie de la réponse sexuelle humaine [7].
En 2006, une étude constate l'action de l'ocytocine pour les contractions involontaires de l'orgasme chez l'homme au moment de l'éjaculation [8]. Une autre étude le prouve chez la femme par relève de la concentration plasmatique d'ocytocine 5 minutes après le coït (la durée de vie de l'ocytocine est très courte) entre les femmes ayant eu un orgasme et les autres non. (2 pg/ml pour les anorgasmisques et 4,6 pg/ml pour les orgasmiques ce qui est un taux sans commune mesure avec les taux nécessaires à la contraction utérine). [9]. Une autre étude (sur les risques de dépression post partum) montre que les femmes enceintes de l'étude, lors d'une analyse de sang entre la 30ème et la 34ème semaine présentent des taux d'ocytocine entre 10 et 250 pg/ml, les plus bas taux suggérant même un risque de dépression post partum. [10].

Extrait d'une thèse de pharmacologie de 2009 : [11]
*« l'**ocytocine** permet le déclenchement des contractions ou leur maintien. L'ocytocine ne peut agir qu'en fin de gestation car, pour agir, il faut que les cellules présentent des*

récepteurs spécifiques qui n'apparaissent qu'en fin de gestation grâce à l'œstradiol et à la PGF2.

L'ocytocine est produite pendant le travail (réflexe neuroendocrinien ou réflexe de Fergusson) par la stimulation de récepteurs situés au niveau du col de l'utérus. La pression exercée par l'enfant sur le col utérin augmente et entraîne l'accroissement de la stimulation des récepteurs qui vont stimuler l'hypothalamus (noyaux para-ventriculaire et supra-optique) et favoriser la sécrétion d'ocytocine. La fixation de l'ocytocine sur les muscles va provoquer l'activation de la PLC (Phospho Lipase C) donc l'augmentation du calcium intracellulaire et la diminution de l'efflux calcique. Il y a donc contraction. Son action se poursuit après l'accouchement et permet la rétraction de l'utérus à sa taille antérieure. ».

Et pour définitivement rassurer la future maman quant aux conséquences négatives d'un possible apport d'ocytocine pour son bébé, nous citerons un mémoire de 2011 de sage-femme [12] sur les autres effets de l'ocytocine produit par la femme enceinte :
« - sur le fœtus : une équipe de chercheurs de l'INSERM a prouvé en 2007 [13] *que l'ocytocine protège le cerveau fœtal en provoquant une diminution de l'activité neuronale pendant l'accouchement. ».*

Dans le domaine plus vaste et voisin qu'est le risque des rapports sexuels en cours de grossesse, nous nous contenterons de citer trois études simplement éclairantes :

- [14]. 210 femmes réparties en deux groupes (108 et 102) pour lesquels le coït était conseillé ou non. L'augmentation de l'activité sexuelle n'a pas augmenté le taux de travail spontané. Néanmoins la fréquence des rapports sexuels était peu différente entre les deux groupes (60,2% vs 39,6%),
- [15]. 574 et 576 femmes respectivement dans les groupes pour lesquels le coït était conseillé ou non. On ne retrouve pas de différence sur le taux d'induction du travail, avec néanmoins une différence de fréquence de rapports sexuels minime entre les deux groupes (85,3% vs 79,9%),
- [16]. les femmes ayant pratiqué un coït sont même moins susceptibles d'avoir un travail spontané avant l'induction du travail prévu. Et de manière générale, le coït et l'orgasme n'ont pas perturbé l'accouchement.

Vers nos patientes qu'il va nous falloir informer il faut le dire en langage simple :

- ce n'est pas l'ocytocine qui enclenche le travail mais la tête du bébé,
- l'ocytocine produite ne peut avoir qu'une action positive sur le fœtus,

- l'effet de l'ocytocine pour la contraction du muscle lisse de l'utérus n'est possible qu'en fin de gestation,
- l'ocytocine des rapports sexuels n'a aucun effet négatif sur la grossesse,
- L'ocytocine des rapports sexuels n'a aucune conséquence significative au cours du travail ; puisque les contractions sont déjà là (et d'une toute autre ampleur) et que la parturiente est déjà à la porte de la salle d'accouchement.

Mais même si on démontre aux femmes enceintes l'absence de risque biologique, le poids des tabous religieux et sociaux déjà posé sur la sexualité en général et sur la sexualité féminine en particulier augmente sensiblement dans cette période de la grossesse où la sexualité procréatrice l'emporte sur la fonction érotique. Ce n'est pas le moment le plus adapté au développement ludique des actes intimes et souvent l'autostimulation superficielle du bouton du clitoris par masturbation (acte déjà ignoré par nombre de femmes [17]) pose particulièrement question dans cette période.

Quant aux autres stimulations du clitoris, celles pratiquées (ou non…) pendant les actes sexuels de la vie privée (par partenaire ou par sex toys…) celles qui concourent à la recherche d'un orgasme, celles du « couple intime » et non du « couple parental », il est nécessaire qu'elles doivent rester du domaine de l'intime pour ne pas compliquer la période de la grossesse déjà suffisamment chargée en émotions, d'autant qu'elles sont parfois en contradiction avec les recommandations sages et efficaces du personnel soignant.

Cet ensemble de données permet aujourd'hui de proposer aux femmes enceintes une méthode qui associe deux concepts complémentaires : Détente Périnéale Externe (DPE © M Bestaux) et Sollicitation des Muscles Ischio caverneux et Bulbo spongieux (SMIB © J Winkel). Cette méthode demande aux femmes de puiser dans leurs capacités analgésiques naturelles latentes sans nécessairement rechercher un plaisir sexuel, sans se focaliser sur le bouton trop excitable du clitoris, sans pénétration interne, sans complicité d'un tiers, sans la recherche d'emblée d'un plaisir sexuel (ignorée par beaucoup rappelons-le) sans positionnement ni fantasmagorie de l'ordre du sexuel et de l'intime, sans imagerie érotique.

Par des actes de massage et une sollicitation de ces muscles, nous espérons enrichir les techniques classiques de préparation à la naissance et participer au suivi gynécologique en complément de toutes les méthodes médicales anti-douleur en œuvre si utilement aujourd'hui. Nous pensons que cette fonction latente du clitoris relève des mécanismes physiologiques du contrôle de la douleur , se basant sur la théorie du gate control [18].

L'effet des stimulations vibratoires transcutanées (SVT) a été étudié en kinésithérapie [19] et utilisé par exemple en odontologie [20].

Par utilisation d'un vibreur (OVD : Outil Vibrant de Détente conforme CE) qui permet une distanciation de l'intime, l'effet analgésique recherché est au cœur du protocole proposé et expliqué comme tel. La parturiente ne se « donne » pas du plaisir (séquence connue ou non de la masturbation jusqu'à l'orgasme). Elle se « donne » les moyens de lutter contre la douleur (séquences prévues dans le cadre de ce protocole). La vibration source de l'OVD évite d'ailleurs le bouton du clitoris (trop « sensible » selon de nombreux témoignages) et l'outil peut être utilisé à travers un drap ou un vêtement. Pour toutes les phases douloureuses à la maison et dans les heures précédant l'accouchement, l'orgasme n'est pas recherché pour lui-même. Ce qui est attendu par la détente de toute la zone périnéale, c'est l'état d'érection des corps caverneux et l'effet analgésique induit par le maintien de cet état, prolongé au mieux dans le temps. En toute objectivité seuls les résultats obtenus avec l'OVD sont comptabilisés, eu égard à la disparité et aux impressions subjectives trop diversifiées des moments d'autostimulation chez les femmes en général.

Le vibreur agit directement sur la zone pubienne entre la symphyse pubienne et le genou du clitoris au niveau de son ligament suspenseur (annexe 1) [21]. C'est le point « ferme » du pubis au dessus du bouton du clitoris, là où le vibreur entre en résonnance plus facilement que dans les zones plus souples des grandes et petites lèvres. Les vibrations obtenues (masselotte dissymétrique sur un axe tournant) sont des fréquences médium et bas-médium semblables aux fréquences sonores des voix humaines. Ni excitation superficielle, ni onde de choc, ni succion des muqueuses, ni pénétration, l'OVD agit par simple massage externe comme ceux pratiqués pour dénouer des articulations ou des muscles douloureux. Il interagit en résonnance, par effet de proximité des ondes ressenties de la source vers les muscles du périnée qui entourent les branches internes du clitoris, de part et d'autre du vagin. Cette stimulation profonde et globale de l'organe clitoris tout entier est très différente de l'excitation superficielle et de surface du bouton du clitoris qui est pratiquée comme jeu sexuel pour une montée vers l'orgasme. Elle crée une phase d'excitation qui se prolonge dans le temps sans nécessité d'orgasme. Cela correspond à la phase de plateau de la réponse sexuelle humaine décrite par Masters et Johnson. [22].

La méthode est applicable même si les contractions utérines potentiellement douloureuses sont rapprochées en phase de travail. La parturiente n'a pas à tourner son esprit, à s'évader honteusement vers l'acte tabou d'une masturbation à dimension sexuée qui lui ferait perdre ses moyens et sa maitrise. Elle reste simplement attentive à sa réalité corporelle, s'adaptant

au mieux au rythme des contractions. La gestion de sa respiration reste possible et complémentaire. Les vibrations de l'OVD s'ajoutent au souffle. Elles sont dans les fréquences rassurantes et habituelles des voix et le résultat est tangible dans une harmonie vibratoire apaisante.

L'érection des corps caverneux du clitoris suppose une détente des muscles qui l'entourent. L'érection n'est pas une contraction ni de l'organe, ni des muscles. L'érection est un afflux sanguin, une libération d'endorphines, et ne peut modifier les contractions involontaires des parois de l'utérus permettant l'ouverture du col. Les forces sollicitées, extrêmement faibles, sont sans commune mesure avec la mise en œuvre des efforts respiratoires demandées pendant la durée du travail ou les forces de poussée à l'œuvre à l'expulsion. Elles n'ont aucun effet sur celles-ci à une telle différence d'échelle.

Plus que des problèmes de logistique ou de sécurité, la faisabilité de la procédure pendant l'accouchement même pose des problèmes éthiques importants, et il est essentiel de les prendre en compte. La nudité (nécessaire), les contraintes médicales et la présence de tiers non informés replacent au moment de l'expulsion et de la délivrance sur l'acte de massage proposé, le voile de la honte et de l'intimité violée [23]. C'est pourquoi une femme ne peut se sentir obligée de pratiquer ce massage à ces moments particuliers. Seule la demande précise et motivée d'une patiente qui voudrait continuer en salle d'accouchement la vibration réussie au cours de la grossesse et du travail pourra être retenue, sous réserve que ce soit l'équipe connue et acceptée par elle, qui l'ait suivie tout au long de la grossesse et qui soit présente à ce moment-là.

2. Objectifs de l'Etude

2.1. Objectif principal

Evaluer l'acceptabilité par la femme enceinte de la recherche de la fonction analgésique du clitoris.

Dans quelle mesure, la recherche de la fonction analgésique de son clitoris est-elle acceptable par la femme enceinte ? Face aux douleurs de la grossesse, comment et de combien la parturiente peut-elle puiser dans sa capacité antidouleur latente et personnelle alors même que le clitoris est plus souvent réservé au seul plaisir sexuel ?

2.2. Objectif secondaire

Évaluer le soulagement de la douleur conséquent à l'utilisation de la fonction analgésique du clitoris.

Si à l'approche ou au moment d'une douleur, la parturiente a voulu et pu faire la stimulation clitoridienne recommandée, la mesure du soulagement obtenu est le deuxième objectif de l'étude pour la période de la grossesse et en tenant compte des moments particuliers du travail, de l'expulsion et de la délivrance.

3. Conception de l'Etude :

3.1. Critères d'évaluations principaux et secondaires

Critère d'évaluation principal :

Parmi les patientes ayant eu la visite de pré-inclusion, proportion de celles qui ont été incluses et qui ont appliqué la procédure au moins deux fois. C'est bien l'acceptabilité de la recherche de la fonction analgésique du clitoris dans ce contexte qui est mesurée.

Critères d'évaluation secondaires :

1/ Perception d'un soulagement d'une douleur chez la femme enceinte par mise en œuvre de la procédure, au moins sur deux tentatives, renseignée par le calendrier FAC (Fonction Analgésique du Clitoris).

L'évaluation est faite par auto-évaluation sur un calendrier exhaustif (calendrier FAC Fonction Analgésique du Clitoris). Cette auto-évaluation, sur papier, est construite sur une échelle numérique de 0 à 10 associée à un visuel reprenant l'échelle visuelle analogique modifiée par le Centre de lutte antidouleur en gynécologie obstétrique du CHU de Rouen (CLuDenGO) [24].

Le soulagement est défini par la case binaire correspondante (oui/non) sur ce calendrier FAC qui est récupéré lors de la visite en post-partum (entre 6 heures et 4 jours après l'accouchement).

2/ taux d'inclusion parmi les patientes après la visite de pré-inclusion (décomposition du critère de jugement principal).

3/ taux d'application de la procédure (au moins deux fois) parmi les femmes incluses (décomposition du critère de jugement principal).

Les épisodes d'auto-stimulation, de stimulation par un tiers ou par des actes sexuels n'entrant pas dans la définition du protocole, ne seront ni évalués ni comptabilisés.

3.2. Description du type de l'Etude

Étude prospective, à un seul groupe (pas de groupe contrôle), mono centrique, pilote.

4. Sélection des personnes de l'Etude

4.1. Critères d'inclusion

- Femme enceinte, quelque soit le terme, primipare ou multipare
- Âge ≥ 18 ans
- Consentement éclairé et écrit de la patiente
- Patiente ayant lu et compris la lettre d'information et signé le formulaire de consentement
- Patiente affiliée à la Sécurité Sociale

4.2. Critères de non-inclusion

- Inaptitude à comprendre le français ou l'information,
- Personne privée de liberté par une décision administrative ou judiciaire
- Personne placée sous sauvegarde de justice
- Personne sous tutelle ou curatelle.

4.3. Modalités de recrutement

L'étude est présentée lors d'une consultation programmée dans le cadre du soin courant par un investigateur (sexologue, une sage-femme ou un médecin). L'investigateur informe la personne et répond à toutes ses questions concernant l'objectif, la nature des contraintes, les risques prévisibles, les bénéfices attendus de la recherche. Il précise également les droits de la personne se prêtant à la recherche dans le cadre de la recherche et remet un exemplaire de la note d'information et du consentement à la personne se prêtant à la recherche.

L'information neutre « Gestion de la douleur, souhaitez-vous participer à la recherche médicale ? » (Annexe 2) peut également avoir été portée à la connaissance de la personne via un affichage en salle de consultation notamment, à remettre du secrétariat de la consultation de gynécologie-obstétrique du CHU si elle est intéressée.

Une consultation d'inclusion avec la sexologue, le médecin ou la sage-femme (V1) sera alors fixée (1er ou 2eme RDV) pour obtenir des réponses adaptées à ses interrogations. Dans le but d'éclairer son consentement éventuel, la patiente peut se faire accompagner d'une personne de son choix (proche, conseil, conjoint, parent).

La consultation d'inclusion (V1) se fait par la sexologue, le médecin ou la sage-femme ayant toute l'expertise pour présenter le projet et l'OVD.
La durée d'inclusion est prévue sur 24 mois.

5. Procédures de l'Etude

5.1. Description de la procédure évaluée lors de la recherche :

La procédure consiste en l'apposition de l'outil thérapeutique vibrant (OVD : Outil Vibrant de Détente) en superficie sur le pubis, entre la symphyse pubienne et le genou du clitoris, au niveau du ligament suspenseur, réalisant un massage de la zone correspondante aux deux branches internes du clitoris. La vibration se propage par la vibration osseuse du pubis vers les branches ischiopubiennes où sont insérés les muscles bulbo-spongieux. Ainsi les muscles ischiocaverneux et bulbo-spongieux (SMIB) qui recouvrent le corps du clitoris sont sollicités. Les corps caverneux du clitoris se mettent en érection, aidant la sécrétion des endorphines attendues, capables de neutraliser en tout ou en partie les douleurs.

L'orgasme féminin n'étant ni le sujet de l'étude, ni nécessaire à l'étude, il est préférable d'éviter le bouton du clitoris en laissant l'OVD en proximité de celui-ci. Mais le fait d'effleurer ou de stimuler plus précisément ce bouton peut déclencher un orgasme, ce qui n'a aucune conséquence négative chez la femme enceinte.

Cet orgasme ainsi déclenché pourra surprendre et étonner une patiente qui n'en a jamais ressenti. Un accompagnement et une bienveillance de la sexologue sera nécessaire pour « débriefer » cette éventuelle première fois. Nul doute que la patiente concernée rectifiera le positionnement légèrement au-dessus de la zone trop sensible (on retrouve facilement ce point précis) pour maintenir l'état bénéfique de décontraction musculaire et se laisser en capacité d'érection de ses corps caverneux.

L'utilisation de cet accessoire permet une distanciation de l'intime d'autant qu'il n'est pas nécessaire de l'utiliser à même la peau. Il est possible de placer l'outil thérapeutique sur la zone indiquée au travers d'un tissu.

Seuls quelques produits peuvent être utilisés pour cette étude et fonctionnent plutôt bien. Tous ont comme point commun une séparation souple et élastique entre le moteur (basse tension) et la masselotte vibrante, ce qui permet un couple moteur bien meilleur. Les ondes longues, prolongées, sans échauffement des muqueuses ni vibration aigue de surface visent à un apaisement des douleurs sans référence obligée à une recherche de plaisir.

Nous avons choisi parmi eux un outil simple qui normalise l'acte (annexe 3). Etant marqué CE, le dispositif dispose d'un certain nombre de garanties d'innocuité, telles que l'absence de substance toxique sur le dispositif ou l'absence de risque électrique ou de radiations.

A l'arrivée d'une douleur, la patiente utilise la procédure et en constate les effets.

5.2. Risques de la procédure:

Les risques sont liés au dispositif OVD. Cet objet conforme CE a une notice qui est lue et remise à la patiente avec l'OVD. Cette notice reprend les bases (comme la non-utilisation en cas de maladie, irritation de la peau ou plaie infectée), en détaillant utilisation et entretien, avertissements et remarques générales et note concernant la protection et l'environnement. (annexe 3)

5.2.1 Réaction allergique au silicone du dispositif OVD

La question d'un antécédent allergique sera systématiquement posée lors de la visite de pré-inclusion et d'inclusion. En cas d'allergie au silicone, le dispositif ne sera pas fourni. L'allergie n'est toutefois pas un critère d'exclusion de l'analyse principale puisque celle-ci porte sur l'acceptabilité.

5.2.2. Risque de corps étranger intra-rectal ou intra-vaginal

L'utilisation du dispositif OVD doit rester à l'extérieur du corps. L'explication est donnée aux patientes intéressées par la recherche lors de la visite de pré-inclusion et d'inclusion, précisée sur la notice d'information et redonnée lors des différentes visites. Ce risque est lié à un mésusage.

5.3. Calendrier FAC (Fonction Analgésique du Clitoris)

Lors de la visite d'inclusion (V1), le calendrier FAC (annexe 4) est délivré à la patiente, à conserver à proximité et à remplir au plus tôt après un épisode douloureux. Si les douleurs abdominales ou périnéales peuvent être assez diffuses voire confuses chez la femme enceinte au cours des mois de grossesse, l'approche du terme et la période du travail lui font identifier plus clairement les douleurs de contractions utérines.

Le calendrier contient une information datée sur la douleur, comprenant le jour, le moment de la journée, le fait que ce soit ou non une contraction (oui / non / ne sait pas) l'intensité de la douleur (0 à 10) avant mise en œuvre de la procédure, l'utilisation ou pas de la procédure, le soulagement de la douleur après utilisation (oui / non) et l'intensité après utilisation. Une

case est dédiée à l'expression libre d'impressions et de commentaires de la part de la patiente. Il est récupéré à la visite du post-partum (V3).

5.4. Traitements/dispositifs/procédures associés interdits dans le cadre du protocole

Aucune procédure ou traitement n'est contre-indiqué dans le cadre de l'étude.

5.5. Traitements/dispositifs/procédures associés autorisés

Toute préparation à la naissance autorisée par le service est possible en complément de l'étude. Le dossier médical est consultable en regard de l'étude.

Au CHU de Rouen, la procédure habituelle d'évaluation de la douleur par une échelle visuelle analogique (EVA) faite par les soignants pendant l'accouchement (infirmiers, sages-femmes, médecins), au rythme d'une évaluation par heure, est maintenue pour les patientes incluses. Le recueil des principaux paramètres de l'accouchement est également adjoint au dossier. Cette somme de résultats permet d'apprécier la cohérence du calendrier FAC et d'affiner l'analyse de chaque cas.

6. Déroulement de l'Etude

6.1. Calendrier de l'étude

- Durée de la période d'inclusion : 24 mois,
- Durée de participation de chaque patient : 9 mois,
- Durée totale de la recherche : 33 mois

6.2. Schéma de la recherche

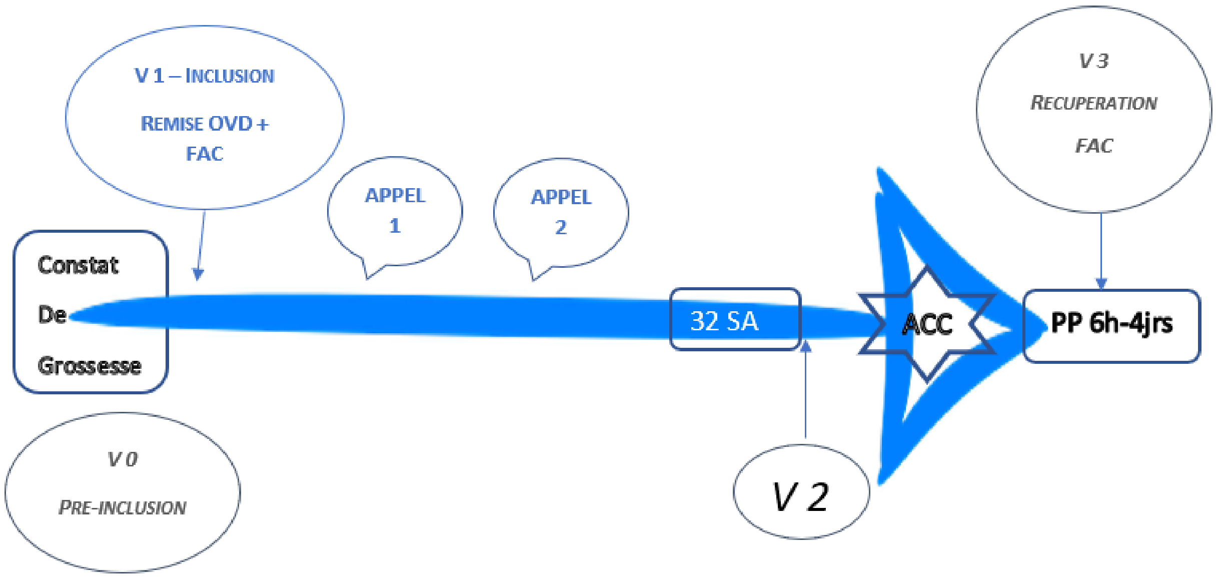

OVD = Outil Vibrant de Détente FAC = (Calendrier) Fonction Antalgique du Clitoris
SA = Semaine d'Aménorrhée ACC = Accouchement PP = Post-partum
APPEL = entretien téléphonique avec la patiente pour vérifier la bonne compréhension de l'utilisation de l'OVD

6.3. Tableau récapitulatif du suivi patient

	Pré-inclusion (sage-femme, sexologue, médecin) V0*	Inclusion V1	Entretien téléphonique (2 semaines après V1 si avant accouchement sinon NA)	Entretien téléphonique (début 3ème trim si en dessous de 37 SA sinon NA)	Visite V2* 32 SA (Si patiente incluse au-delà de 32 SA, visite NA)	Acc	Visite V3 * post-partum
Information de la patiente	✓	✓					
Consultation d'inclusion		✓					
Vérification des critères d'inclusion		✓					
Recueil du Consentement éclairé		✓					
Remise de l'outil thérapeutique OVD ₮		✓					
Délivrance du calendrier FAC ¶		✓					
Vérification de la bonne utilisation de l'OVD ₮			✓	✓	✓	✓	
Récupération du calendrier FAC ¶							✓

*Visite programmée dans le cadre du parcours de soin
₮ OVD = Outil Vibrant de Détente
¶ FAC = Fonction Antalgique du Clitoris

6.4. Visite de pré-inclusion - Visite V0

L'étude est présentée lors d'une consultation programmée dans le cadre du soin courant par un investigateur (sexologue, une sage-femme ou un médecin). L'investigateur informe la personne et répond à toutes ses questions concernant l'objectif, la nature des contraintes, les risques prévisibles, les bénéfices attendus de la recherche. Il précise également les droits de la personne se prêtant à la recherche dans le cadre de la recherche et remet un exemplaire de la note d'information et du consentement à la personne se prêtant à la recherche.

6.5. Visite d'inclusion – V 1

L'investigateur (sexologue, une sage-femme ou un médecin). informe la patiente sur la nature de l'étude, de la part de recherche qui est attendue d'elle, son objectif, ses risques et contraintes, les bénéfices attendus.

Cette visite doit permettre :

- de l'aider à comprendre dans quel registre se situe cette recherche de la fonction analgésique par massage vibrant (la fonction analgésique n'est pas la fonction érotique ou la fonction sexuelle)
- de différencier la stimulation du bouton du clitoris à visée sexuelle vers l'orgasme et la procédure proposée.
- d'informer la patiente sur les données anatomiques acquises (schémas tirés de la vidéo d'anatomie 3D du périnée féminin Université de Lyon [21])
- de décrire la procédure (lecture exhaustive de l'article 5.1 ci-dessus).
- lui permettre de mieux comprendre et cadrer la pratique proposée par rapport à ses pratiques corporelles, sexuelles personnelles et conjugales.
- de comprendre les risques (lecture exhaustive du paragraphe 5 .2)

Cet échange d'informations est indispensable pour éviter les contresens, amalgames, confusions, raccourcis qui ne manquent jamais de polluer tout discours ou projet soignant dès que le mot « clitoris » apparaît dans une conversation. Ce « cher inconnu » du film du

même nom [25] doit l'être un peu moins à l'issue de cette visite afin d'éclairer le consentement de la patiente, surtout si cette patiente n'a jamais ressenti d'orgasme.

Le bon usage du dispositif est expliqué à la patiente, ainsi que les risques liés au mésusage.

L'allergie au silicone est recherchée (déclaration de la patiente). En cas d'allergie, l'acceptabilité pourra être évaluée mais le dispositif ne sera pas remis à la patiente et elle ne sera donc pas concernée par les étapes suivantes.

La patiente formule toutes ses questions.

L'investigateur a la responsabilité de vérifier les critères d'inclusion de celle-ci. Il recueille le consentement libre de la patiente et s'assure que ce consentement est éclairé des informations déjà fournies à V0.

Il précise les conditions des épisodes de massage et de la poursuite du protocole en regard des limites que la patiente souhaite fixer à la découverte possible de sa propre intimité. Pour établir un climat de confiance et de sérénité, il définit avec elle la confidentialité du cadre dans lequel elle souhaite participer à l'étude pendant les épisodes particuliers où cette intimité pourrait être limitée (à l'hôpital). Pour le moment du travail il rassure la patiente sur la confidentialité du lieu et par rapport à l'équipe soignante (pas toujours au fait de l'étude).

Pour la suite de l'accouchement (expulsion et délivrance) et seulement dans le cas où la patiente choisirait de poursuivre le protocole, il lui confirme la présence très nécessaire d'un ou des deux investigateurs du projet.

Si la patiente donne son accord de participation, la patiente et l'investigateur inscrivent leurs noms et prénoms, datent et signent le formulaire de consentement, dont une copie remise à la patiente et l'original conservé par l'investigateur.
Un numéro d'appel téléphonique ainsi qu'une adresse mail dédiée sont fournis à la patiente pour tout renseignement pendant la durée de l'étude. Un numéro de téléphone sera demandé pour vérifier le bon fonctionnement et utilisation du disposition OVD par un entretien téléphonique au début du dernier trimestre de la grossesse.

l'outil vibrant de détente OVD est remis à la patiente.

La procédure est anatomiquement précisée. Les conditions d'utilisation (hygiène, durée) sont explicitées par la lecture de la notice (annexe 3) donnée avec l'objet. L'utilisation de l'OVD est à nouveau explicitée avec un schéma remis à la patiente, tiré de la vidéo sur le périnée féminin pour le positionnement exact (annexe 1).

Les 2 appels téléphoniques ainsi que la visite V2 sont optionnels. Si possible et selon le terme de la grossesse, une date est prise pour les entretiens téléphoniques.
Le calendrier FAC est remis et expliqué à la patiente.

6.6. Suivi par appel téléphonique (2 semaines après V1 si avant accouchement sinon NA)

La patiente est contactée par la sexologue pour la vérification du bon fonctionnement de l'OVD et l'assurance de sa bonne utilisation.

6.7. Suivi par appel téléphonique (début du 3ème trimestre si en dessous de 37 SA sinon NA)

La patiente est contactée par la sexologue pour la vérification du bon fonctionnement de l'OVD et l'assurance de sa bonne utilisation.

6.8. Visite de suivi – V2 (Si patiente incluse au-delà de 32 SA, visite NA)

Cette visite V2 est prévue à la 32ème semaine d'aménorrhée afin de vérifier la compréhension de remplissage du calendrier FAC et répondre aux questions éventuelles, notamment sur les conditions de confidentialité de la recherche.

6.9. Visite de fin de l'Etude (dernière visite réalisée par la patiente) – V3

Cette visite est prévue en post-partum, dans les semaines suivant l'accouchement pour la récupération du calendrier FAC.

6.10. Règles d'arrêt de l'Etude

- *Arrêt prématuré, définitif ou temporaire, de l'ensemble de l'Etude*

L'Etude peut être suspendue ou arrêtée par le promoteur ou à la demande du Comité de Protection des Personnes (CPP) à tout moment.

- *Pour la patiente incluse, arrêt prématuré, définitif ou temporaire, de l'Etude*

Un arrêt prématuré de la participation de la patiente à l'étude pourra être acté pour les motifs suivants :

- en cas d'interruption de grossesse, volontaire ou involontaire,
- en cas d'événement imprévisible ou indésirable contre-indiquant la poursuite de l'étude
- par décision de l'investigateur dans l'intérêt de la patiente,
- en cas de patiente perdue de vue,
- en cas de décès de la patiente.

Tout arrêt prématuré sera daté et sa raison sera renseignée par l'investigateur.
La patiente n'aura plus de visite dans le cadre de l'étude à l'exception des visites de suivi d'éventuels événements indésirables.
En cas d'événement indésirable ayant justifié l'arrêt prématuré de l'étude, des visites supplémentaires de suivi, adaptées à l'événement, seront effectuées. L'information sur l'évolution de l'événement indésirable grave sera renseignée pour l'étude.

- *Par la patiente incluse, arrêt prématuré, définitif ou temporaire, de l'étude*

La participation à l'étude n'impliquant pas une utilisation « obligatoire » de l'outil, ni un protocole « imposé » lors de séances « impératives » de massage, la simple non-observance du programme de recherche de la fonction analgésique du clitoris par elle-même n'est pas un motif d'arrêt. Il est seulement noté sur le calendrier FAC cette non-observance et comptabilisée comme tel.

Mais toute patiente souhaitant se retirer formellement de l'étude quel qu'en soit le motif pourra retirer son consentement à tout moment et en informer l'investigateur qui doit mettre tout en œuvre pour :

- Consigner la décision de la patiente par écrit,
- Identifier le ou les motifs de sortie d'étude et les reporter dans le CRF.

Ce retrait n'a pas d'incidence sur l'utilisation des données obtenues sur la base du consentement éclairé exprimé avant que celui-ci n'ait été retiré.

7. Sécurité des personnes

C'est une étude mentionnée au 2° de l'article L.1121-1 du code de la Santé Publique avec risques et contraintes minimes et le référentiel applicable est le système de vigilance sanitaire (Article L.1123-10 du Code de la Santé Publique).

Les risques potentiels suivants pour la personne attendus sont les suivants :

- réaction allergique au silicone du dispositif OVD
- corps étranger intra-rectal ou intra-vaginal en cas de mésusage du dispositif OVD

<u>Gestion des Evènement Indésirables</u>

L'investigateur recueille les évènements indésirables dans le formulaire de recueil des événements indésirables situé dans le CRF électronique :

- L'investigateur évalue chaque événement indésirable au regard de sa gravité.
- L'investigateur évalue l'intensité des événements indésirables observés chez le participant à la recherche et de la reporter dans le cahier d'observation, soit en s'aidant d'une échelle de gradation des événements indésirables annexée au protocole (exemple la classification NCI-CTCAE version 5.0 (Novembre 2017) ou de Clavien-Dindo pour les essais de cancérologie), soit par des termes plus généralistes comme :

Léger (grade 1)	n'interfère pas avec l'activité quotidienne habituelle
Modéré (grade 2)	limite partiellement l'activité quotidienne habituelle
Sévère (grade 3)	limite l'activité quotidienne habituelle
Très sévère (grade 4)	menace le pronostic vital
Décès (grade 5)	

- L'investigateur évalue le lien de causalité des événements indésirables le dispositif faisant l'objet de la recherche

8. Statistiques :

8.1. Description des méthodes statistiques

L'analyse principale sera faite par estimation du pourcentage de femmes ayant appliqué la procédure au moins deux fois parmi celles qui ont eu la visite d'information.
D'un point de vue statistique, il ne sera pas possible de conserver des informations détaillées sur les femmes venues à la visite d'information mais non incluses ; par contre le volume de visites d'informations pourra être évalué précisément par enregistrement du nombre de visites.

Un pourcentage supérieur à 25% sera considéré comme acceptable.

La proportion de sujets ayant appliqué au moins une fois la procédure quel que soit le moment, parmi l'ensemble des femmes inclus sera évaluée en intention de traiter. Ainsi, un sujet présentant un calendrier incomplet sera analysé sur la base des données disponibles.
En l'absence de donnée, on considèrera que la procédure n'a pas été appliquée.

Une analyse de sécurité, portant sur les événements indésirables sera faite par simple description des effets indésirables et de leur nombre. Des regroupements d'événements seront effectués si nécessaire, afin de présenter un tableau synthétique.

8.2. Nombre prévu de personnes à inclure dans l'Etude

Étant donné qu'il s'agit d'une étude de faisabilité, les seules données de la littérature étant des cas rapportés très isolés, il est préférable de réaliser l'étude sur un petit échantillon pilote. Avec 64 patientes venant à la visite d'information, 32 incluses et 16 utilisant la procédure, l'incertitude sur le taux 16/64 correspondra à un facteur 2,5 entre la borne haute et la borne basse de l'intervalle de confiance.
C'est-à-dire, l'intervalle de confiance attendu est (15% à 37%).

8.3. Degré de signification prévu

L'analyse principale sera l'estimation d'un intervalle de confiance bilatéral à 95% d'un pourcentage. Il n'y a pas de degré de signification.

8.4. Critères statistiques d'arrêt de l'Etude

L'étude sera arrêtée lorsque les 32 patientes dont l'inclusion est prévue auront atteint leur visite finale V3, sauf si des difficultés d'inclusion venaient à interrompre prématurément l'étude.

8.5. Modalités de remplacement

Aucun remplacement n'est prévu.

8.6. Réalisation d'une analyse intermédiaire

Aucune analyse intermédiaire d'efficacité ni de sécurité n'est planifiée. En cas d'événement indésirable, l'étude peut néanmoins être interrompue prématurément, si la sécurité des patientes est compromise.

9. Droit d'accès aux données et documents source.

Le promoteur est chargé d'obtenir l'accord de l'ensemble des parties impliquées dans l'étude afin de garantir l'accès direct à tous les lieux de déroulement de l'étude, aux données sources, aux documents sources et aux rapports dans un but de contrôle de qualité et d'audit par le promoteur.

Les investigateurs mettront les documents et les données individuelles strictement nécessaires au suivi, au contrôle qualité et à l'audit de recherche impliquant la personne

humaine, à la disposition des personnes ayant un accès à ces documents conformément aux dispositions réglementaires et législatives en vigueur.

Les données source sont définies comme tout document original permettant de prouver l'existence ou l'exactitude d'une donnée enregistrée au cours de l'essai. Conformément aux dispositions législatives en vigueur (articles L.1121-3 et R.5121-13 du code de la santé publique), les personnes ayant un accès direct aux données sources prendront toutes les précautions nécessaires en vue d'assurer la confidentialité des informations relatives au matériel utilisé, à l'étude, aux personnes qui s'y prêtent et notamment en ce qui concerne leur identité ainsi qu'aux résultats obtenus. Ces personnes, au même titre que les investigateurs eux-mêmes, sont soumises au secret professionnel.

Pendant l'étude impliquant la personne humaine ou à son issue, les données recueillies sur les personnes qui s'y prêtent et transmises au promoteur par les investigateurs (ou tous autres intervenants spécialisés) seront rendues anonymes. Elles ne doivent en aucun cas faire apparaître en clair les noms des personnes concernées ni leur adresse. Seul le professionnel de santé qui dirige la réalisation de l'étude peut conserver le lien entre l'identité codée des personnes se prêtant à la recherche et leurs prénoms et noms.

Le code d'identification anonymisé des patients consistera en un numéro chronologique d'inclusion associé à la première lettre du nom de famille et à la première lettre du prénom.
Le promoteur s'assurera que chaque personne qui se prête à l'étude a été informée de ses droits et des modalités d'exercice de ceux-ci. De même, il s'assurera que chaque personne qui se prête à l'étude a donné son accord par écrit pour l'accès aux données individuelles la concernant et strictement nécessaires au contrôle de qualité de l'étude.

Dans le cadre de l'Etude, un traitement informatique de certaines données personnelles des professionnels de santé, conformément à la MR001, sera aussi mis en œuvre pour permettre la mise en place et le déroulement de l'Etude. A cette fin, ces données personnelles seront transmises au Promoteur ou aux personnes ou sociétés agissant pour son compte, en France. Ces données pourront également, dans des conditions assurant leur confidentialité, être transmises aux autorités de santé française ou étrangères et à d'autres entités du promoteur.

Cette Etude relève de la méthodologie de référence MR-001, relative à la gestion et la transmissions des données recueillies au cours de l'étude. Le promoteur de cette étude a reçu de la part de la CNIL une déclaration de conformité à cette méthodologie de référence.

Conformément aux dispositions de la loi relative à l'informatique, aux fichiers et aux libertés, les professionnels de santé disposent à tout moment d'un droit d'accès et de rectification des données informatisées les concernant. Ils disposent également d'un droit d'opposition à la transmission des données couvertes par le secret professionnel susceptibles d'être utilisées dans le cadre de cette étude et d'être traitées.

10. Contrôle et assurance qualité

10.1. Gestion des données relatives à l'étude

10.1.1. Saisie et stockage des données

Lors de leur recueil, les informations concernant les patients participant à cette étude seront anonymisées selon les recommandations établies par la CNIL.

Les données cliniques seront saisies dans la base de données sécurisée en ligne développée par le CHU de Rouen en utilisant le logiciel Clinsight® (Ennov Group, 251 rue du Faubourg Saint Martin à Paris 75010 - France). Les données seront hébergées sur un serveur sécurisé localisé à la Direction du Système d'Information du CHU de Rouen. Des copies de sauvegarde du contenu de ce serveur sont réalisées quotidiennement.

Les données seront saisies par le médecin investigateur ou le Technicien d'Etude Clinique, qui disposa de comptes personnels et sécurisés (identifiant et mot de passe personnalisé à 6 caractères minimum) pour l'accès à la base de données de l'étude. Les personnes réalisant le traitement informatique (biostatisticien) auront accès aux données une fois la base gelée par la mise à disposition de celle-ci sur un serveur sécurisé accessible uniquement au gestionnaire de données et au biostatisticien de l'étude. La saisie des données sera réalisée, sous la responsabilité de l'investigateur, par un membre de son équipe (TEC, infirmière,…).

10.1.2. Modalités de traitement, vérification et validation des données

La saisie étant réalisée, des contrôles de bornes seront programmés en fonction des tests de cohérence programmés par le gestionnaire de données. Une validation des données sera réalisée pour l'analyse statistique, et des demandes de correction seront émises à l'investigateur ou au TEC de l'étude, qui s'engage à compléter et corriger les données en conséquence.

10.1.3. Destinataire des données traitées

<u>Les catégories des personnes suivantes ont accès aux données traitées :</u>

- Le responsable de traitement et les personnes agissant pour son compte ;

- Le responsable scientifique (investigateur coordonnateur) de l'étude ;
- Les professionnels de santé intervenant dans la recherche et les personnels agissant sous leur responsabilité (investigateur, IRC, TEC du centre investigateur, etc.) ;
- Les personnes responsables de l'assurance de qualité, c'est-à-dire chargées de contrôler et d'évaluer la qualité et l'authenticité des données collectées ;
- Les personnels d'autorités sanitaires et d'autorités publiques de contrôle légalement habilités, dans le cadre d'une mission particulière ou de l'exercice d'un droit de communication (ANSM, FDA, CNIL, etc.) ;
- Les personnes chargées des analyses statistiques ;
- Les personnes chargées des affaires réglementaires et de l'enregistrement de la recherche auprès des autorités compétentes
- Les personnels habilités agissant sous la responsabilité de l'organisme d'assurance garantissant la responsabilité civile du promoteur.

L'ensemble de ces catégories de personnes sont soumises au secret professionnel.
Les données peuvent être transmises à des partenaires contractuels, sous une forme qui ne doit pas permettre l'identification directe ou indirecte des personnes se prêtant à la recherche. La présentation des résultats du traitement de données ne peut en aucun cas permettre l'identification directe ou indirecte des personnes se prêtant à l'étude.

Le promoteur en tant que responsable du traitement met en œuvre des procédures permettant qu'il soit donné suite à toutes demandes (accès, rectification, opposition) des personnes se prêtant à l'étude.

10.2. Contrôle Qualité

L'Attaché de Recherche Clinique, mandaté par le promoteur, fera des visites de façon régulière du centre de l'étude selon le plan de monitoring établi en début d'étude lors :

- de la mise en place de l'étude,
- en cours d'étude selon le rythme des inclusions,
- en fin d'étude.

L'attaché de Recherche Clinique devra s'assurer du respect des droits et de la sécurité des sujets, de la fiabilité, de la qualité et de la traçabilité des données et des informations transmises, et de vérifier que l'étude est conduite en conformité avec le protocole, les BPC et le dispositif réglementaire et législatif en vigueur.
Les visites auront donc pour objet de vérifier et valider :

- les données des cahiers d'observation définies dans le plan de monitoring de la recherche
- l'éligibilité des patients inclus : respect des critères d'inclusion et de non-inclusion
- le respect des procédures d'information des patients et recueil de leurs consentements
- le respect des procédures spécifiques du protocole, du calendrier FAC, de la liste LPC, du suivi du patient
- la qualité des données recueillies dans le cahier d'observation ou Case Report Form (CRF) : exactes, complètes et cohérentes
- le respect des procédures de déclaration des EIG et faits nouveaux
- la bonne gestion et traçabilité des dispositifs de l'étude (comptabilité des dispositifs OVD).

A l'issue de chaque visite, sera rédigé un rapport de monitoring standardisé par l'ARC qui sera revu par le promoteur.

10.3. Audit et inspection

Un audit peut être réalisé à tout moment par des personnes mandatées par le promoteur et indépendantes des responsables de l'étude. Il a pour objectif de s'assurer de la qualité de l'étude, de la validité de ses résultats et du respect de la loi et des réglementations en vigueur. Les investigateurs acceptent de se conformer aux exigences du promoteur et à l'Autorité compétente en ce qui concerne un audit ou une inspection de l'essai.
L'audit pourra s'appliquer à tous les stades de l'étude, du développement du protocole à la publication des résultats et au classement des données utilisées ou produites dans le cadre de l'étude.

11. Conservation et archivage des données relatives à l'Etude

Le promoteur et les investigateurs archivent les documents essentiels et les données relatifs qui leur sont spécifiques à cette étude conformément aux Bonnes Pratiques Cliniques **pour une durée de 25 ans** suivant la fin de l'étude.

Les documents essentiels à archiver sont :

- Le protocole et les amendements éventuels au protocole
- Les cahiers d'observation
- Les dossiers sources des participants ayant signé un consentement (uniquement pour l'investigateur)
- Tous les autres documents essentiels et courriers relatifs à la recherche, notamment le classeur investigateur.

La conservation et l'archivage de tous ces documents respectifs sont sous la responsabilité de l'investigateur et du promoteur pendant la durée réglementaire d'archivage. Aucun déplacement ou destruction ne pourra être effectué sans l'accord du promoteur. Au terme de la durée réglementaire d'archivage, le promoteur sera consulté pour destruction. Toutes les données, tous les documents et rapports pourront faire l'objet d'audit ou d'inspection.

12. Considérations éthiques et réglementaires

Le promoteur, l'investigateur et la co-investigatrice s'engagent à ce que cette étude soit réalisée en conformité avec la loi n°2012-300 du 5 Mars 2012 relative au recherches impliquant la personne humaine et ses versions consolidées ainsi qu'en accord avec la loi n°2004-806 du 9 août 2004 et ses versions consolidées, les Bonnes Pratiques Cliniques (I.C.H. dans sa version consolidée et décision du 24 novembre 2006) et la déclaration d'Helsinki version d'octobre 2008 (qui peut être retrouvée dans sa version intégrale sur le site http://www.wma.net).

L'étude est conduite conformément au présent protocole. Hormis dans les situations d'urgence nécessitant la mise en place d'actes thérapeutiques précis, l'investigateur et la co-investigatrice s'engagent à respecter le protocole en tous points en particulier en ce qui concerne le recueil du consentement et la notification et le suivi des événements indésirables. Cette étude a reçu l'avis favorable du Comité de Protection des Personnes (CPP) de *nom du CPP*.

Le CHU de ROUEN, promoteur de cette étude, a souscrit un contrat d'assurance en responsabilité civile auprès de la société Biomedic Insure conformément aux dispositions de l'article L1121-10 du code de la santé publique.

Les données enregistrées à l'occasion de cette étude font l'objet d'un traitement informatisé au CHU de Rouen dans le respect de la loi n°78-17 du 6 janvier 1978 relative à l'informatique, aux fichiers et aux libertés modifiée par la loi 2004-801 du 6 août 2004 et ses versions consolidées.

Cette étude entre dans le cadre de la « Méthodologie de référence » (MR-001) en application des dispositions de l'article 54 alinéa 5 de la loi du 6 janvier 1978 modifiée relative à l'information, aux fichiers et aux libertés. Ce changement a été homologué par décision du 5 janvier 2006. Le CHU de ROUEN a souscrit auprès de la CNIL un engagement de conformité à cette « Méthodologie de référence ».
Cette étude est enregistrée sur le site http://clinicaltrials.gov/

Amendement au protocole : Un amendement est une modification substantielle.

Toute modification substantielle, c'est à dire toute modification de nature à avoir un impact significatif sur la protection des personnes, sur les conditions de validité et sur les résultats de la recherche, sur la qualité et la sécurité des outils expérimentés, sur l'interprétation des documents scientifiques qui viennent appuyer le déroulement de la recherche ou sur les modalités de conduite de celle-ci, fait l'objet d'un amendement écrit qui est soumis au promoteur ; celui-ci doit *en fonction du type de modification) :*

- Soit obtenir, préalablement à sa mise en œuvre, un avis favorable du CPP,
- *Soit être transmis pour information au CPP*

Tous les amendements au protocole doivent être portés à la connaissance de l'investigateur et la co-investigatrice, qui s'engagent à en respecter le contenu en signant un document d'adhésion en cas de modification du protocole.

Tout amendement qui modifie la prise en charge des patients ou les bénéfices, risques et contraintes de la recherche fait l'objet d'une nouvelle note d'information et d'un nouveau formulaire de consentement dont le recueil suit la même procédure que celle précitée.

13. Règles relatives à la publication et à la communication.

L'analyse des résultats fera l'objet de communications dans les congrès et de publications.

Le texte des publications et des communications sera discuté avec l'ensemble des investigateurs participants à l'essai. Les co-auteurs sont l'investigateur principal et la co-investigatrice, le Professeur Eric VERSPYCK et le Docteur Marie-Madeleine, Manon BESTAUX-BRETHEZ. Les règles de publication sont les suivantes :

En français :

Centre Hospitalier Universitaire (CHU) de Rouen, service de Gynécologie-Obstétrique, Rouen, F 76000, France.

Centre Hospitalier Universitaire (CHU) de Rouen, unité de Biostatistique, Rouen, F 76000, France.

En anglais :

Rouen University Hospital, department of Obstetrics & Gynaecology, Rouen, F 76000, France.

Rouen University Hospital, department of Biostatistics, Rouen, F 76000, France.

14. Références à la littérature scientifique

1. Schick V., Herbenick D, Jozkowski KN, Jawed-Wessel S, Reece M. The sexual consumer: characteristics, expectations, ans experinences of women attending in -home sex toy parties. J Sex Marital Ther. 2013;39(2) : 160-75.
2. Meston CM, Frohlich PF. The Neurobiology of Sexual Function. Arch Gen Psychiatry. 2000 Nov 1;57(11):1012–30.
3. O'Connell HE, Hutson JM, Anderson CR, Plenter RJ. Anatomical relationship between urethra and clitoris. J Urol. 1998 Jun;159(6):1892–7.
4. Foldes P, Buisson O. The clitoral complex: a dynamic sonographic study. J Sex Med. 2009 May;6(5):1223–31.
5. Whipple B, Komisaruk BR. Analgesia produced in women by genital self-stimulation. J Sex Res. 1988 Jan;24(1):130–40.
6. Vuillet Anna. La masturbation aurait-elle un rôle analgésique à jouer dans l'accouchement ? (Phases de travail et d'expulsion) [Travail de Fin d'Etudes en vue de l'obtention du grade de Bachelier SAGE-FEMME]. [Angleur (BE)]: Haute Ecole Libre Mosane Sainte-Julienne; 2018.
7. Carmichael MS, Humbert R, Dixen J, Palmisano G, Greenleaf W, Davidson JM. Plasma oxytocin increases in the human sexual response. J Clin Endocrinol Metab. 1987 Jan;64(1):27-31.
8. Thackare H1, Nicholson HD, Whittington K. Oxytocin--its role in male reproduction and new potential therapeutic uses. 2006 Jul-Aug;12(4):437-48. Epub 2006 Jan 25.
9. Caruso S1,2, Mauro D1,2, Scalia G3, Palermo CI3, Rapisarda AMC1,2, Cianci A1,2 Oxytocin plasma levels in orgasmic and anorgasmic women Gynecol Endocrinol. 2018 Jan;34(1):69-72. doi: 10.1080/09513590.2017.1336219. Epub 2017 Jun 11
10. Skrundz M[1], Bolten M, Nast I, Hellhammer DH, Meinlschmidt G. Plasma oxytocin concentration during pregnancy is associated with development of postpartum depression.Neuropsychopharmacology.2011Aug;36(9):1886-93. doi:10.1038/npp.2011.74. Epub 2011 May 11.
11. Isabelle Lacroix Thèse de doctorat en pharmacologie Pharmacovigilance chez la femme enceinte : aspects maternel et néonatal (exemple des substances psychoactives) Toulouse 2009 http://thesesups.ups-tlse.fr/579/1/Lacroix_Isabelle.pdf
12. Claire Gricourt. Le travail dirigé par ocytocine de synthèse au CHU de Rouen. Étude rétrospectiveet unicentrique. À propos de 169 cas. Gynécologie et obstétrique. 2011. dumas-00631182 https://dumas.ccsd.cnrs.fr/dumas-00631182
13. Tyzio R[1], Nardou R, Ferrari DC, Tsintsadze T, Shahrokhi A, Eftekhari S, Khalilov I, Tsintsadze V, Brouchoud C, Chazal G, Lemonnier E, Lozovaya N, Burnashev N, Ben-Ari Y Oxytocin-mediated GABA inhibition during delivery attenuates autism

pathogenesis in rodent offspring.Science. 2014 Feb 7;343(6171):675-9. doi: 10.1126/science.1247190.

14. Peng Tan;Choon Yow;Siti Omar. doi.org/10.1097/01.AOG.0000267201.70965.ec Effect of Coital Activity on Onset of Labor in Women Scheduled for Labor Induction: A Randomized Controlled Trial Obstetrics & Gynecology. 110(4):820-826, OCT 2007
15. NS Omar , PC Tan , N Sabir , ES Yusop , SZ Omar Coitus to expedite the onset of labour: a randomised trial General obstetrics 2012 https://doi.org/10.1111/1471-0528.12054
16. Peng Tan;Anggeriana Andi;Noor Azmi;M Noraihan Effect of Coitus at Term on Length of Gestation, Induction of Labor, and Mode of Delivery Obstetrics & Gynecology. 108(1):134-140, JULY 2006 DOI: 10.1097/01.AOG.0000223229.83920.af
17. Bajos N, Bozon M, Beltzer N. Enquête sur la sexualité en France: pratiques, genre et santé. Editions La Découverte; 2008.
18. Ronald Melzack et Patrick D. Walles The gate control theory of pain, 1965. https://doi.org/10.1016/S1082-3174(96)80050-X .

19 Roll Jean-Pierre, Stimulation vibratoire transcutanée et douleur, 2019 https://doi.org/10.1016/j.douler.2019.09.004

20. Hegde KM, R N, Srinivasan I, D R MK, Melwani A, Radhakrishna S, Effect of vibration during local anesthesia administration on pain, anxiety, and behavior of pediatric patients aged 6-11 years: A crossover split-mouth study, J Dent Anesth Pain Med. 2019 Jun ;19(3):143-149. doi: 10.17245/jdapm.2019.19.3.143. Epub 2019 Jun 30
21. Patrice THIRIET Institut des Sciences et Techniques de la Réadaptation - Université Lyon 1 vidéo d’anatomie 3D du périnée féminin Université de Lyon, 2015 https://www.youtube.com/watch?v=9psQO8RNfXw
22. William.H. Masters et Virginia. E. Johnson, *Human Sexual Response*, Little, Brown and Co, 1966 (ISBN 0-3165-4987-8).
23. Postel T. Naissance et jouissance : mise en évidence de l’existence d’un orgasme obstétrical. Sexologies. 2013 Oct 1;22(4):165–8.
24. Bonnet S, Caltero C, Dessogne F, Fanchette M-P, Fawer K, Ferchichi R, et al. Naissance d’une échelle visuelle analogique. 14ème congrès de la SFETD; 2014 Nov 22; Toulouse.
25. Film "Le clitoris ce cher inconnu" 2003 - Michèle Dominici, Variety Moszinski et Stephen Firmin Hum Reprod Update.

15. Liste des abréviations

SA : Semaine d'Aménorrhée

FAC : Fonction Analgésique du Clitoris

ACC : Accouchement

APPEL : Entretien téléphonique en début de 3ème trimestre

OVD : Outil Vibrant de Détente

CE : Conformité aux normes européennes

PP : Post-partum

16. Liste des annexes :

- Annexe 1 = photo positionnement OVD, selon photo tirée de la vidéo de l'Université de Lyon sur le périnée féminin en 3D [16],

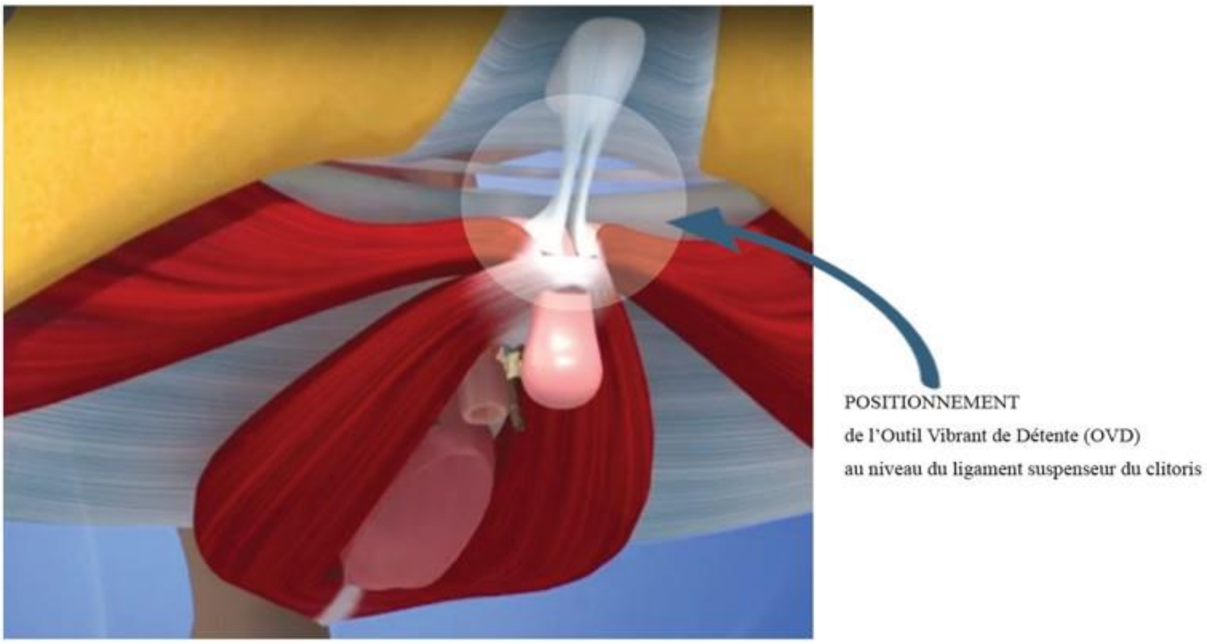

- Annexe 2 = document donné à toutes les patientes enceintes à la première consultation de constat de grossesse,

# - GESTION DE LA DOULEUR -
Souhaitez-vous participer à la recherche médicale ?

Le CHU de Rouen mène actuellement un projet de recherche médicale sur le soulagement des douleurs obstétricales pendant la grossesse et lors de l'accouchement,
par une méthode non médicamenteuse.

Si vous souhaitez des renseignements complémentaires avant de décider d'y participer,
merci de prendre rendez-vous au secrétariat du service de gynécologie et d'obstétrique
pour une consultation d'information avec le Dr Manon BESTAUX,
Pavillon Martainville, 02 32 88 84 22

Vous pouvez vous faire accompagner d'une personne de votre choix.

- Annexe 3 = Outil OVD (outil vibrant de détente) marqué CE.

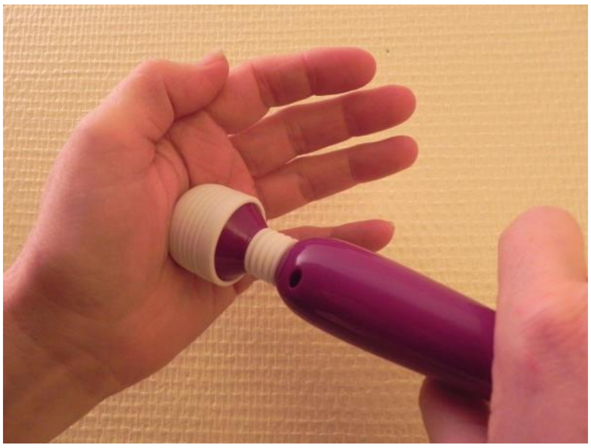

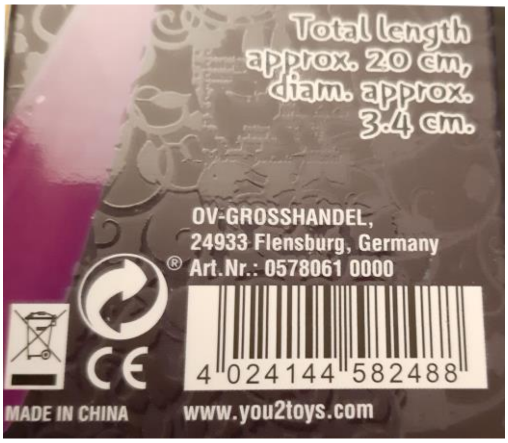

Notice d'utilisation et d'entretien du lovetoy vibrant

1. Nettoyez le lovetoy vibrant à l'eau chaude et au savon avant la première et après chaque nouvelle utilisation. Après chaque utilisation anale, l'appareil doit être nettoyé en profondeur avant de pouvoir être utilisé au niveau vaginal.
   Soyez prudents – n'oubliez pas qu'il s'agit d'un appareil électrique, l'humidité dans le compartiment à piles peut causer des dommages.
2. Appliquez assez de lubrifiant sur la surface du lovetoy vibrant. Veillez en ce faisant à ce que l'unité de contrôle fonctionnant avec des piles ne soit pas endommagée, car cela pourrait entraîner un mauvais fonctionnement de l'appareil. ***IMPORTANT :*** n'utilisez que du lubrifiant soluble à l'eau ! Les lubrifiants à base d'huile attaquent les matériaux.
3. Après l'avoir posé ou introduit en douceur, appuyez ou tournez avec précaution le contrôle de vitesse et sélectionnez la vitesse de vibration souhaitée ou choisissez une fonction de l'unité de contrôle qui correspond à vos souhaits. Tournez, appuyez ou poussez le régulateur de vitesse lentement et en douceur, jamais violemment.
4. Eteignez le lovetoy vibrant après utilisation et retirez-le doucement de votre corps.
5. Avant de le ranger, laissez refroidir le lovetoy vibrant après utilisation.
6. Gardez le lovetoy vibrant au propre, au frais et au sec, car l'humidité peut endommager les piles et le moteur. Protéger des rayons du soleil !
7. Rangez votre lovetoy vibrant séparément et pas avec des articles composés de matières différentes afin d'éviter des réactions chimiques.

Avertissements et remarques générales :

1. Conserver hors de la portée des enfants. Les petites pièces peuvent être avalées.
2. Ne pas utiliser le lovetoy vibrant en cas de maladie ou d'irritation de la peau ou sur une peau infectée.
3. Afin d'éviter les maladies et les infections, ne transmettez pas le lovetoy vibrant à un tiers.
4. Veillez respecter les notes relatives aux matériaux indiquées sur l'emballage afin d'éviter les réactions allergiques, comme par ex. au latex entre autres.
5. Assurez-vous que les piles sont bien placées correctement dans le compartiment à piles. Vérifiez les polarités + et – et placez les piles dans le bon sens.
6. Lorsque le lovetoy vibrant est imperméable, vérifiez que le compartiment est fermé hermétiquement après avoir placé les piles.
7. En cas de surchauffe du lovetoy vibrant, éteignez-le immédiatement et laissez-le refroidir avant de l'utiliser à nouveau.
8. N'essayez pas de recharger des piles non rechargeables.
9. Ne jetez pas les piles dans le feu.
10. Ne pliez, ni ne détendez pas trop le Lovetoy. N'employez pas la force.
11. N'utilisez le lovetoy vibrant que pour accompagner des préliminaires partagés, comme appareil de massage ou de stimulation, pour la masturbation ou pour accompagner les rapports sexuels et ne l'utilisez pas dans d'autres buts.
12. Le fournisseur n'assume aucune responsabilité pour l'article, si celui-ci n'est pas utilisé correctement.
13. Le produit n'est pas destiné à un usage professionnel.

Note concernant la protection de l'environnement :

ne pas jeter les emballages, ni les appareils usagés, mais les amener aux points de collecte. Veuillez remettre les appareils usagés conformément à votre législation locale et aux directives 2002/96/EG et 2006/66/EG aux points de collectes respectifs.
Le sigle de la poubelle à roulette barrée (🗑) signifie que cet appareil doit être remis à un centre de collecte pour appareils électroménagers usagés afin d'assurer le meilleur recyclage des matières premières.

Retour des piles

La place des piles n'est pas dans la poubelle de la maison. En tant qu'utilisateur, vous êtes légalement tenus de ramener les piles usagées, par exemple dans les déchetteries publiques ou là où les piles vous ont été vendues. Les piles des catégories mentionnées ici peuvent nous être renvoyées gratuitement. Les piles contenant des substances toxiques portent le signe 🗑 et l'un des symboles chimiques Cd (la pile contient du cadmium), Hg (la pile contient du mercure) ou Pb (la pile contient du plomb). Les piles au lithium et les piles rechargeables de tous les systèmes ne doivent être rendues au centre de collecte que déchargées. La polarité de ce type de piles doit être recouverte de bande adhésive avant d'être remises au centre de collecte pour éviter les courts-circuits provoqués en cas de non déchargement total des piles.

- Annexe 4 = Calendrier FAC

Nom :
Prénom :

Mois :

Protocole FAC - CHU de Rouen

Jour du mois	Moment de douleur				Contraction			Intensité de la douleur * (entre 1 et 10)	Utilisation procédure		SI UTILISATION PROCEDURE			Impressions ou Commentaires (facultatif)
											soulagement		Intensité de la douleur *	
	Matin	Après Midi	Soir	Nuit	Oui	Non	Ne sait pas		Oui	Non	Oui	Non		
Ex : 10	X						X	7	X		X		2	*heureusement j'étais chez moi*

intensité de la douleur :

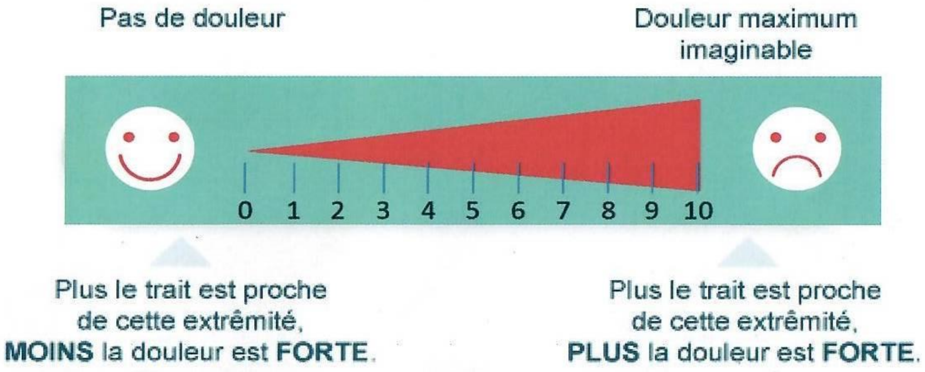

Supplement: S1 File — (PDF) [file pone.0333112.s001.pdf]
