# Supplementary material for: Potential analgesic function of the clitoris in pregnant women: A feasibility study
Source: PLoS One. 2025 Dec 9;20(12):e0333112. doi: 10.1371/journal.pone.0333112 (PMC12688119; doi:10.1371/journal.pone.0333112)
Supplement: S2 File — (PDF) [file pone.0333112.s002.pdf]

CONFIDENTIAL

STUDY PROTOCOL INVOLVING THE HUMAN PERSON MENTIONED IN 2° OF ARTICLE L.1121-1 OF THE CSP

Clitoris potential Analgesic Function research by pregnant women : *C.A.F.*

2018/0415/HP

2018-A03483-52

Version 3 of 28/06/2021

Principal Investigator:
Professor Eric VERSPYCK,
Department of Obstetrics and Gynaecology, Rouen University Hospital
1 rue de Germont 76031 Rouen Cedex
Tel.: 02 32 88 82 44

Co-Investigator:
Dr Marie-Madeleine, Manon BESTAUX-BRETHEZ
Department of Obstetrics and Gynaecology
Rouen University Hospital
1 rue de Germont 76031 Rouen Cedex
Tel.: 02 32 88 82 44

Promoter:
Delegation for Clinical Research and Innovation,
Rouen University Hospital
1 rue de Germont 76031 Rouen Cedex
Tel.: 02 32 88 82 65
Fax: 02 32 88 82 87

Methodologist and Biostatistician:
Dr André GILLIBERT
Biostatistics and Methodology Unit, Rouen University Hospital
1 rue de Germont
76031 Rouen Cedex
Phone : 02.32.88.84.94

Synopsis

**Study Title**<br>**Code Sponsor**	Clitoris potential Analgesic Function research by pregnant women (C.A.F.): Feasibility Study<br>2018/0415/HP
**Principal Investigator**	Professor Eric VERSPYCK,<br>Department of Obstetrics and Gynaecology<br>CHU de Rouen, 1 rue de Germont 76031 Rouen cedex
**Classification**	Study protocol involving the human person mentioned in 2° of Article L.1121-1 of the CSP
**Promoter**	Delegation for Clinical Research and Innovation Rouen University Hospital, 1 rue de Germont 76031 Rouen Cedex Tel.: 02 32 88 82 65 Fax: 02 32 88 82 87
**Population concerned**	Pregnant women who understand French
**Objectives of the study**	**Main Objective** :<br>Evaluation of the acceptability by the pregnant woman of the search for the clitoris potential analgesic function.<br><br>**Secondary Objectives** :<br>To assess pain relief resulting from the activation of the clitoris analgesic function.
**Evaluation Criteria**	**Primary endpoint** :<br>Of the patients who had the pre-inclusion visit, proportion of those who were included and performed the procedure at least twice<br><br>**Secondary endpoints** :<br>1/ Perception of pain relief in pregnant women by implementation of the procedure, at least on two attempts, indicated by the CAF (Clitoris Analgesic Function) calendar.<br>2/ Inclusion rate among patients after the pre-inclusion visit (decomposition of the primary outcome).<br>3/ Rate of application of the procedure (at least twice) among the women included (decomposition of the primary outcome)
**Type of study**	Prospective, single-group (no control group), single-center, pilot study
**Inclusion Criteria**	• Pregnant woman, whatever the term, primiparous or multiparous<br>• Age ≥ 18 years<br>• Patient who has read and understood the information letter and signed the consent form<br>• Patient affiliated to the Social Security

**Criteria non-inclusion**	• Inability to understand French or information,<br>• Person deprived of liberty by an administrative or judicial decision<br>• Person placed under judicial protection<br>• Person under tutorship or curatorship
**Search Procedure**	In case of pain, massage by apposition of the OVD vibrating therapeutic Device (Vibrating Relaxation Device in accordance with CE) between the pubic symphysis and the knee of the clitoris on the suspensory ligament, for the relaxation of the ischiocavernosus and bulbo cancellous muscles, allowing the erection of the corpora cavernosa of the clitoris. Assessment by the CAF (Clitoris Analgesic Function) calendar provided by the patient.
**Theoretical number of patients to be included**	32
**Anticipated number of Centres**	1
**Statistical Data Analysis**	The main analysis will be done by estimating the percentage of women who performed the procedure at least twice among those who had the pre-inclusion visit.<br>From a statistical point of view, it will not be possible to keep detailed information on women who came to the pre-inclusion visit but were not included; On the other hand, the volume of visits can be accurately assessed by recording the number of visits. A percentage above 25% will be considered acceptable.<br><br>Since this is a feasibility study, the only data in the literature being very isolated reported cases, it is preferable to conduct the study on a small pilot sample. With 64 patients coming to the information visit, 32 included and 16 using the procedure, the uncertainty on the 16/64 rate will correspond to a factor of 2.5 between the upper and lower bound of the confidence interval.<br>That is, the expected confidence interval is (15% to 37%).
**Provisional timetable**	Length of Inclusion Period = 24 months<br>With about 56 women giving birth per week, counting 1/20 who will consult at the sexual pre-inclusion visit, or 2.8 women per week. With an inclusion rate of 50% of these women, the rate of inclusion will be 1.4 per week. It will then take 23 weeks, or about 24 months, for inclusion.<br>Duration of follow-up per patient: maximum 9 months Number of Visits: 3<br><br>Total duration: 33 months

Summary

Synopsis **2**
Summary **4**
1. Scientific rationale and general description of the Study **6**
2. Objectives of the Study **12**
2.1. Primary Objective 12
2.2. Secondary Objective 12
3. Study Design **13**
3.1. Primary and Secondary Endpoints 13
3.2. Description of the type of study 14
4. Selection of the persons to include **14**
4.1. Inclusion Criteria 14
4.2. Non-inclusion criteria 14
4.3. Recruitment procedures 14
5. Study Procedures **15**
5.1. Description of the procedure evaluated during the search 15
5.2. Risks of the procedure 16
5.3. CAF Calendar (Clitoris Analgesic Function) 16
5.4. Associated Treatments/Devices/Procedures Prohibited as Part of the Protocol 17
5.5. Associated Treatments/Devices/Procedures Allowed 17
6. Conduct of the Study **17**
6.1. Study Timeline 17
6.2. Research Diagram CALL 18
6.3. Summary table of patient follow-up 18
6.4. Pre-Inclusion Tour - V0 Tour 19
6.5. Inclusion Visit – V1 19
6.6. Follow-up by phone call (2 weeks after V1) 21
6.7. Follow-up by phone call (beginning of 3rd trimester) 21
6.8. Follow-up Visit – V2 21
End of Study Visit (Last Visit Performed by the Patient) – V3 21
6.9. Rules for Stopping the Study 21
7. Personal Safety **22**
Management of Undesirable Events 23
8. Statistics **23**
8.1. Description of Statistical Methods 23
8.2. Expected number of people to be included in the Study 24
8.3. Expected Materiality 24
8.4. Statistical Criteria for Study Discontinuation 24
8.5. Replacement Arrangements 24
8.6. Performing an interim analysis 24
9. Right of access to source data and documents **24**
10. Quality Control & Assurance **26**
10.1. Study Data Management 26
10.1.1. Data entry and storage 26
10.1.2. Data processing, verification and validation 26
10.1.3. Recipient of the processed data 26
10.2. Quality control 27
10.3. Audit & Inspection 28
11. Retention and archiving of data relating to the Study **28**
12. Ethical and Regulatory Considerations **29**

13. Rules on publication and communication. 30
14. References to scientific literature...31
15. List of abbreviations 33
16. List of Appendices 34

1. Scientific rationale and general description of the Study

For women, pregnancy is a physiological phenomenon that can be accompanied, during its evolution over time, by painful abdominal or perineal clinical manifestations, including "false" uterine contractions throughout pregnancy or "true" contractions as term approaches. As a first-line treatment, non-medication is recommended to manage pain during this period. The clitoris analgesic function has its place in this context, provided that the parturient intends to draw the expected analgesic benefits from it, and then is willing and able to do so.

Outside of pregnancy, in sexology consultations, testimonies abound on the improvement of dyspareunia and the fight against vaginismus by stimulation of the clitoris before and during sexual intercourse. They document a large number of sexual acts that are more or less effective, such as self-stimulation of the clitoris button or foreplay performed by the partner. The use of props in the context of sex education to improve sex life, and in particular to reduce pain, is recommended, as shown by a 2012 study of more than 2500 adult women in long-term monogamous relationships [1]. It is the analgesic function of the clitoris that is sought. In the phase of sexual arousal, an analgesic state related to the erection of the corpora cavernosa of the clitoris allows and facilitates sexual intercourse. Endorphins and cortisol are released into the body for a relaxing and disinhibiting effect [2].

Prior to these sexual acts, we hypothesize an analgesic function linked to the overall reproductive function that gives a more useful place to the clitoris in the female reproductive system. Nature doesn't please for pleasure's sake, so it would be surprising if the clitoris wasn't there for nothing.

The clitoris is not always represented in the genital tract. In fact, it is practically excluded from the female reproductive system. The commonly accepted function of the clitoris is that of being exclusively reserved for female sexual pleasure, even if it is difficult to explain why such a large minority of women do not know this "pleasure function". Knowledge of female anatomy is unequally shared by women. Clitoris self-stimulation is nowhere near as systematic as male masturbation. The female orgasm also seems to be more difficult to access.

Wanting too much to talk about sexual pleasure in the very particular context of pregnancy directly confronts intimate barriers and often throws an additional disturbance (psychological, cultural or social) into an already delicate period. We might as well place ourselves on the side of knowledge and allow each patient to be an enlightened actor in her own birth.

Helen O'Conell's recent and documented anatomical knowledge [3]. Odile Buisson and Pierre Foldès [4] define the clitoris as an organ in its own right in the female genital tract. It is finally represented in its entirety in a textbook. Its branches enclose the vagina. The ischio cavernosus and bulbo cancellous muscles are involved in the control of the corpora cavernosa of the clitoris and may or may not allow them to erect. It is no longer objectively the "small" button, the "small" penis, the "small trigger". But his image does not improve: he continues to be immediately associated, attached and by analogy to the masculine accompanied by the sexual taboo par excellence: masturbation.

The lack of scientific data on the functions of the clitoris is a problem of history and interpretation: how to study an organ that has not been anatomically described for centuries and, correlatively, how to study a physiological function different from the imposed idea (exclusive organ of female sexual pleasure) since this one-sided interpretation is based on the same anatomical ignorance?

For women who have an enlightened knowledge of their anatomy (through self-stimulation), it is often reported that masturbation is a proven technique for reducing period pain as well as lower back pain during pregnancy.

Whipple *et al* proved in 1988, on a small series of cases, that genital stimulation of women raised pain detection and tolerance thresholds [5]. Healthy female volunteers, aged 26 to 57, were recruited. Vaginal and clitoral stimulation increased the threshold for detection of pain caused by standardized compression (up to 1 kg) of the fingers of the left hand. The small number of cases is fortunate in view of the method used, as the provocation of pain is fortunately no longer part of the ethical methods accepted today. It should be noted that this is masturbation (self-stimulation) and an anatomical confusion that is now outdated (between clitoris and vagina).

A Belgian midwifery thesis, presented in 2018, describes testimonies of stimulation of the clitoris during painful moments of childbirth that cause the pain to drop in a way that is

[6]. These reported cases were identified by a midwifery student who recruited them from a Facebook group advocating home birth with about 5,000 members. The invitation message stated that women were looking for women who felt the urge or need to masturbate to relieve pain during childbirth. Only women who did not have an epidural were included. Five testimonies from women describing pain relief from uterine contractions during childbirth were collected in qualitative telephone interviews.
There is clearly a very strong selection bias and this remains only reported cases.

It should be noted that this is a masturbation of women who know their bodies well enough and under such particular conditions, in voluntary interruption of hospital care, that these testimonies must be given their sole quality as testimonies.

None of the women interviewed had or sought orgasm as if only the erection phase was to be taken into account. The term "orgasmic" childbirth is therefore inappropriate for these referenced cases.

To get a clearer picture and to properly inform our patients, we detail the example of oxytocin, a contracting hormone in pregnant women, which is declared everywhere as the common hormone and the link between woman and mother, childbirth and pleasure.

Already in 1987, it was proven that oxytocin is part of the human sexual response [7]. In 2006, a study found the action of oxytocin for involuntary orgasm contractions in men at the time of ejaculation [8]. Another study proves this in women by increasing the plasma concentration of oxytocin 5 minutes after coitus (the lifespan of oxytocin is very short) between women who have had an orgasm and those who have not. (2 pg/ml for anorgasmics and 4.6 pg/ml for orgasmics, which is a level out of all proportion to the levels necessary for uterine contraction). [9]. Another study (on the risks of postpartum depression) shows that pregnant women in the study, during a blood test between 30 and 34 weeks, have oxytocin levels between 10 and 250 pg/ml, the lowest levels even suggesting a risk of postpartum depression. [10]

Excerpt from a 2009 pharmacology thesis: [11]
*"**Oxytocin** is used to trigger or maintain contractions. Oxytocin can only act at the end of gestation because, in order to work, the cells must have specific receptors that only appear at the end of gestation thanks to estradiol and PGF2.*

Oxytocin is produced during childbirth work (neuroendocrine reflex or Ferguson's reflex) by stimulating receptors in the cervix. The pressure exerted by the child on the cervix increases

and leads to an increase in the stimulation of receptors that stimulate the hypothalamus (paraventricular and supraoptic nuclei) and promote the secretion of oxytocin. The binding of oxytocin to the muscles will cause the activation of PLC (Phospho Lipase C) and therefore the increase of intracellular calcium and the decrease of calcium efflux. So there is a contraction. Its action continues after childbirth and allows the uterus to retract to its previous size. "I am not sure

And to definitively reassure the mother-to-be about the negative consequences of a possible intake of oxytocin for her baby, we will quote a 2011 memoir by a midwife [12] on the other effects of oxytocin produced by pregnant women:
"- on the fetus: a team of researchers from INSERM proved in 2007 [13] *that oxytocin protects the fetal brain by causing a decrease in neuronal activity during childbirth."*

In the broader and related field of the risk of sexual intercourse during pregnancy, we will confine ourselves to citing three studies that are simply enlightening:

- [14]. 210 women divided into two groups (108 and 102) for whom coitus was advised or not. The increase in sexual activity did not increase the rate of spontaneous work. Nevertheless, the frequency of sexual intercourse differed little between the two groups (60.2% vs. 39.6%),
- [15]. 574 and 576 women, respectively, in groups for which coitus was advised or not. There was no difference in the rate of induction of childbirth work, although there was a minimal difference in the frequency of sexual intercourse between the two groups (85.3% vs. 79.9%).
- [16]. Women who have performed coitus are even less likely to have spontaneous labor before induction of the intended labor. And generally speaking, coitus and orgasm did not disturb the delivery.

To our patients, whom we will have to inform, it must be said in plain language:

- it is not oxytocin that initiates childbirth work but the baby's head,
- the oxytocin produced can only have a positive effect on the fetus,

- the effect of oxytocin for the contraction of the smooth muscle of the uterus is only possible at the end of gestation,
- oxytocin from sexual intercourse has no negative effect on pregnancy,
- Oxytocin from sexual intercourse has no significant consequences during work; since the contractions are already there (and of a completely different magnitude) and the parturient is already at the door of the delivery room.

But even if pregnant women are shown the absence of biological risk, the weight of the religious and social taboos already imposed on sexuality in general and on female sexuality in particular increases significantly in this period of pregnancy when procreative sexuality prevails over erotic function. This is not the most suitable time for the playful development of intimate acts and often the superficial self-stimulation of the clitoris button by masturbation (an act already ignored by many women [17]) is particularly problematic in this period.

As for the other stimulation of the clitoris, those practiced (or not...) during sexual acts in private life (by partner or by sex toys, etc.), those that contribute to the search for an orgasm, those of the "intimate couple" and not of the "parental couple", it is necessary that they remain in the realm of intimacy so as not to complicate the period of pregnancy, which is already sufficiently emotionally charged, especially since they are sometimes in contradiction with the wise and effective recommendations of the nursing staff.

This set of data now makes it possible to offer pregnant women a method that combines two complementary concepts: External Perineal Relaxation (Bestal M EPD ©) and Solicitation of the Cavernous Ischio and Cancellous Bulbo Muscles (SMIB © J Winkel). This method asks women to tap into their latent natural analgesic capacities without necessarily seeking sexual pleasure, without focusing on the overly excitable button of the clitoris, without internal penetration, without the complicity of a third party, without the search for sexual pleasure from the outset (ignored by many, let's not forget) without positioning or phantasmagoria of the order of the sexual and the intimate, without erotic imagery.

By massaging and soliciting these muscles, we hope to enrich the classic techniques of preparation for birth and participate in gynecological follow-up in addition to all the pain-relieving medical methods that are so useful today. We believe that this latent function of the clitoris is part of the physiological mechanisms of pain control, based on the theory of gate control [18].

The effect of transcutaneous vibrational stimulation (TST) has been studied in physiotherapy [19] and used, for example, in dentistry [20].

By using a vibrator (OVD: One Vibrating Device, in CE compliant) which allows a distancing of the intimate, the analgesic effect sought is at the heart of the proposed protocol and explained as such. The parturient does not "give" herself pleasure (known or unknown sequence from masturbation to orgasm). It "gives" itself the means to fight against pain (sequences provided for in the framework of this protocol). The source vibration of the OVD also avoids the clitoris button (too "sensitive" according to many testimonies) and the tool can be used through a sheet or a piece of clothing. For all painful phases at home and in the hours leading up to childbirth, orgasm is not sought for its own sake. What is expected by the relaxation of the entire perineal area is the state of erection of the corpora cavernosa and the analgesic effect induced by the maintenance of this state, prolonged at best over time. Objectively, only the results obtained with the OVD are counted, given the disparity and the overly diverse subjective impressions of moments of self-stimulation in women in general.

The vibrator acts directly on the pubic area between the pubic symphysis and the knee of the clitoris at the level of its suspensory ligament (Appendix 1) [21]. It is the "firm" point of the pubis above the clitoris button, where the vibrator resonates more easily than in the softer areas of the labia majora and minora. The resulting vibrations (asymmetrical weight on a rotating axis) are mid and low-mid frequencies similar to the sound frequencies of human voices. Neither superficial arousal, nor shock wave, nor suction of the mucous membranes, nor penetration, OVD acts by simple external massage like those practiced to untie painful joints or muscles. It interacts in resonance, by the proximity effect of the waves felt from the source to the muscles of the perineum that surround the internal branches of the clitoris, on either side of the vagina. This deep, all-encompassing stimulation of the entire clitoris organ is very different from the superficial, surface arousal of the clitoris button that is practiced as a sexual game for a rush to orgasm. It creates a phase of arousal that lasts over time without the need for orgasm. This corresponds to the plateau phase of the human sexual response described by Masters and Johnson. [22].

The method is applicable even if the potentially painful uterine contractions are close together during labor. The parturient does not have to turn her mind, to escape shamefully to the taboo act of masturbation with a sexual dimension that would make her lose her means and her control. She simply remains attentive to her bodily reality, adapting

at best at the rhythm of contractions. Breathing management remains possible and complementary. The vibrations of the OVD add to the breath. They are in the reassuring and usual frequencies of the voices and the result is tangible in a soothing vibratory harmony.

The erection of the corpora cavernosa of the clitoris requires a relaxation of the muscles around it. An erection is not a contraction of either the organ or the muscles. An erection is a blood flow, a release of endorphins, and cannot alter the involuntary contractions of the walls of the uterus allowing the opening of the cervix. The forces involved, which are extremely low, are out of all proportion to the implementation of the respiratory forces required during the duration of the work or the pushing forces at work at the expulsion. They have no effect on them at such a difference in scale.

More than logistical or safety issues, the feasibility of the procedure during childbirth itself poses significant ethical issues, and it is essential to take them into account. The (necessary) nudity, the medical constraints and the presence of uninformed third parties place the veil of shame and violated intimacy on the proposed act of massage at the time of expulsion and delivery [23]. That's why a woman can't feel compelled to practice this massage at these particular times. Only the precise and motivated request of a patient who would like to continue the successful vibration in the delivery room during pregnancy and labor can be retained, provided that it is the team known and accepted by her, who has followed her throughout the pregnancy and who is present at that time.

2. Objectives of the Study

2.1. Primary Objective

To assess the acceptability by the pregnant woman of the search for the clitoris analgesic function.

To what extent is the search for the clitoris analgesic function acceptable to the pregnant woman? Faced with the pains of pregnancy, how and how much can the parturient draw on her latent and personal pain-relieving capacity when the clitoris is more often reserved for sexual pleasure alone?

2.2. Secondary Objective

To assess pain relief resulting from the use of the clitoris analgesic function.

If, at the approach or time of pain, the parturient was willing and able to perform the recommended clitoral stimulation, the measurement of the relief obtained is the second objective of the study for the period of pregnancy and taking into account the particular moments of work, expulsion and delivery.

3. Study Design:

3.1. Primary and Secondary Endpoints

Primary endpoint :

Of the patients who had the pre-inclusion visit, proportion of those who were included and who performed the procedure at least twice. It is the acceptability of the search for the clitoris analgesic function in this context that is measured.

Secondary endpoints:

1/ Perception of pain relief in pregnant women by implementation of the procedure, at least on two attempts, indicated by the CAF (Clitoris Analgesic Function calendar.

The assessment is done by self-assessment on a comprehensive schedule (CAF Clitoris Analgesic Function Calendar). This self-assessment, on paper, is based on a numerical scale from 0 to 10 associated with a visual based on the visual analogue scale modified by the Centre for Pain Control in Obstetrics and Gynaecology of the University Hospital of Rouen (CLuDenGO) [24].

Relief is defined by the corresponding binary box (yes/no) on this CAF schedule that is retrieved at the postpartum visit (between 6 hours and 4 days postpartum).

2/ Inclusion rate among patients after the pre-inclusion visit (decomposition of the primary outcome).

3/ Rate of application of the procedure (at least twice) among the women included (decomposition of the primary outcome).

Episodes of self-stimulation, third-party stimulation, or sexual acts that do not fall within the scope of the protocol will not be evaluated or counted.

3.2. Description of the type of study

Prospective, single-group (no control group), single-center, pilot study.

4. Selection of the persons to include

4.1. Inclusion Criteria

- Pregnant woman, whatever the term, primiparous or multiparous
- Age≥ 18 years
- Informed and written consent of the patient
- Patient who has read and understood the information letter and signed the consent form
- Patient affiliated to the Public Health Insurrances

4.2. Non-inclusion criteria

- Inability to understand French or information,
- Person deprived of liberty by an administrative or judicial decision
- Person placed under judicial protection
- Person under tutorship or curatorship.

4.3. Recruitment procedures

The study is presented during a consultation scheduled as part of routine care by an investigator (sexologist, midwife or doctor). The investigator informs the person and answers all questions about the objective, the nature of the constraints, the foreseeable risks, the expected benefits of the research. It also sets out the rights of the research-consenting person in the course of the research and provides a copy of the briefing note and consent to the research-consenting person.

Neutral information "Pain management, do you want to participate in medical research?" (Appendix 2) may also have been brought to the attention of the person via a notice in the consultation room, in particular, to be handed over from the secretariat of the gynaecology-obstetrics consultation of the CHU if he or she is interested.

An inclusion consultation with the sexologist, doctor or midwife (V1) will then be scheduled (1st or 2nd appointment) to obtain answers adapted to their questions. In order to clarify her possible consent, the patient may be accompanied by a person of her choice (relative, counselor, spouse, parent).

The inclusion consultation (V1) is done by the sexologist, doctor or midwife with all the expertise to present the project and the OVD.
The duration of inclusion is expected to be 24 months.

5. Study Procedures

5.1. Description of the procedure evaluated during the research:

The procedure consists of the placement of One Vibrating Device therapeutic (OVD : One Vibrating Device) on the surface of the pubis, between the pubic symphysis and the knee of the clitoris, at the level of the suspensory ligament, performing a massage of the area corresponding to the two inner branches of the clitoris. The vibration propagates through the bony vibration of the pubis to the ischiopubic branches where the bulbosponous muscles are inserted. Thus, the ischiocavernosus and bulbospongiosus muscles (IBMS) that cover the body of the clitoris are solicited. The corpora cavernosa of the clitoris become erect, helping the secretion of the expected endorphins, capable of neutralizing all or part of the pain.

Since the female orgasm is neither the subject of the study nor necessary for the study, it is best to avoid the clitoris button by leaving the OVD close to it. But touching or stimulating this button more precisely can trigger an orgasm, which has no negative consequences for the pregnant woman.

This orgasm may surprise and astonish a patient who has never felt one. The support and benevolence of the sexologist will be necessary to “debrief” this possible first time. There is no doubt that the patient concerned will rectify the positioning slightly above the overly sensitive area (this precise point is easily found) to maintain the beneficial state of muscle relaxation and leave her corpora cavernosa in the capacity for erection.

The use of this accessory allows a distancing from the intimate, especially since it is not necessary to use it next to the skin. It is possible to place the therapeutic tool on the indicated area through a tissue.

Only a few products can be used for this study and work pretty well. What they all have in common is a flexible and elastic separation between the motor (low voltage) and the vibrating weight, which allows for a much better motor torque. Long, prolonged waves, without heating the mucous membranes or acute surface vibration, aim to soothe pain without any obligatory reference to a search for pleasure.

We have chosen from among them a simple tool that standardizes the act (Appendix 3). Being CE marked, the device has a number of safety guarantees, such as the absence of toxic substances on the device or the absence of electrical hazards or radiation.

When pain occurs, the patient uses the procedure and sees its effects.

5.2. Risks of the procedure:

The risks are related to the OVD device. This CE-compliant object has a leaflet that is read and given to the patient along with the OVD. This leaflet covers the basics (such as non-use in case of illness, skin irritation or infected wound), detailing use and care, warnings and general remarks and a note concerning protection and the environment. (Appendix 3)

5.2.1 Allergic reaction to OVD device silicone

The question of an allergic history will be systematically asked during the pre-inclusion and inclusion visit. In case of silicone allergy, the device will not be provided. However, allergy is not an exclusion criterion from the main analysis as it focuses on acceptability.

5.2.2. Risk of Intrarectal or Intravaginal Foreign Body

The use of the OVD device should be kept outside the body. The explanation is given to patients interested in the research during the pre-inclusion and inclusion visit, specified on the information leaflet and given again during the various visits. This risk is related to misuse.

5.3. CAF Calendar (Clitoris Analgesic Function)

At the inclusion visit (V1), the CAF schedule (Appendix 4) is issued to the patient, to be kept nearby and completed as soon as possible after a painful episode. If abdominal or perineal pain can be quite diffuse or even confused in pregnant women during the months of pregnancy, the approach of term and the period of labor make them identify more clearly the pain of uterine contractions.

The calendar contains dated information about the pain, including the day, time of day, whether or not it is a contraction (yes/no/don't know), pain intensity (0-10) before the procedure is implemented, whether or not the procedure is used, pain relief after use (yes/no), and the intensity after use. One case is dedicated to the free expression of impressions and comments on the part of the patient. It is recovered at the postpartum visit (V3).

5.4. Associated Treatments/Devices/Procedures Prohibited as Part of the Protocol

No procedures or treatments are contraindicated in the study.

5.5. Associated Treatments/Devices/Procedures Allowed

Any preparation for birth authorized by the service is possible in addition to the study. The medical record is available next to the study.

At Rouen University Hospital, the usual procedure of assessing pain by a visual analogue scale (VAS) carried out by caregivers during childbirth (nurses, midwives, doctors), at the rate of one assessment per hour, is maintained for the included patients. The collection of the main parameters of childbirth is also attached to the file. This sum of results makes it possible to assess the coherence of the CAF calendar and to refine the analysis of each case.

6. Conduct of the Study

6.1. Study Timeline

- Length of inclusion period: 24 months,
- Duration of participation of each patient: 9 months,
- Total duration of the search: 33 months

6.2. Research Diagram

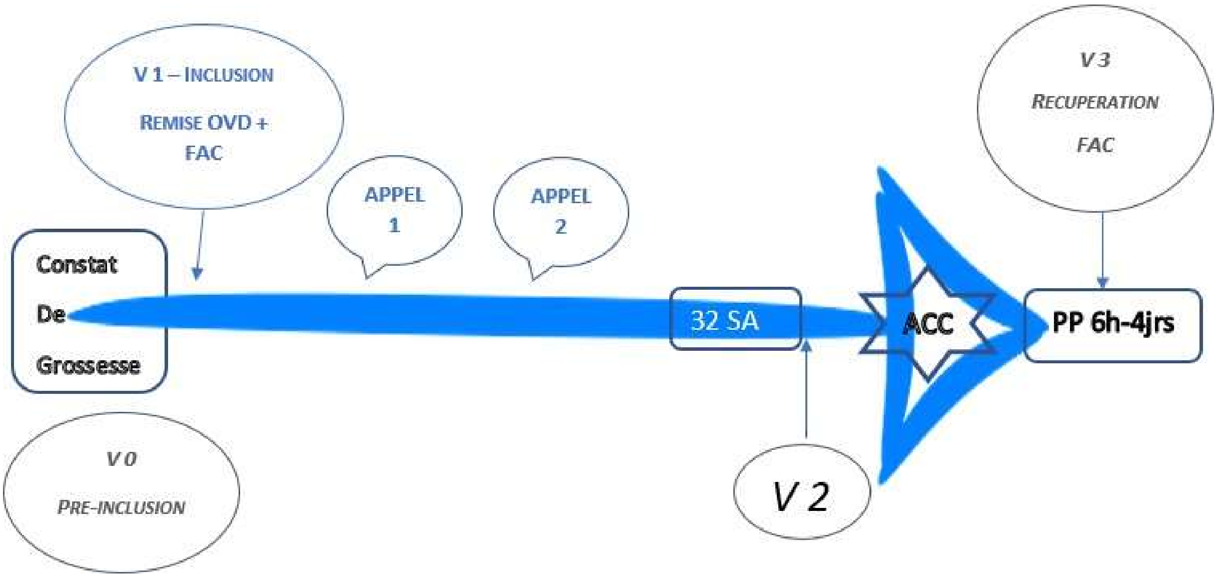

OVD = One Vibrating Device   CAF = (Calendar) Clitoris Analgesic function
AS = amenorrhea week   ACC = Childbirth PP = Postpartum
APPEL = call = interview with the patient to verify understanding of the use of OVD

6.3. Summary table of patient follow-up

	Pre-Inclusion (midwife, sexologist), Physician) V0*	Inclusion V1	Telephone interview (2 weeks after V1 if before delivery otherwise NA)	Telephone interview (beginning of the 3rd trim if below 37 SA otherwise NA)	Visit V2* 32 SA (If patient included beyond 32 weeks, NA visit)	Acc	Visit V3 * postpartum
Patient Information	✓	✓					
Inclusion Consultation		✓					
Verification of Inclusion Criteria		✓					
Collection of Informed Consent		✓					
Delivery of the OVD₮ Therapeutic		✓					
Issuance of the CAF Calendar ¶		✓					
Verification of the correct use of the OVD₮			✓	✓	✓	✓	
Retrieving the CAF Calendar ¶							✓

*Visit scheduled as part of the care pathway
₮ OVD = One Vibrating Device
¶ CAF = Clitoris Analgesic Function

6.4. Pre-Inclusion Visit - V0 Visit

The study is presented during a consultation scheduled as part of routine care by an investigator (sexologist, midwife or doctor). The investigator informs the person and answers all questions about the objective, the nature of the constraints, the foreseeable risks, the expected benefits of the research. It also sets out the rights of the research-consenting person in the course of the research and provides a copy of the briefing note and consent to the research-consenting person.

6.5. Inclusion Visit – V1

The investigator (sexologist, midwife or doctor). informs the patient about the nature of the study, the amount of research that is expected of her, its objective, its risks and constraints, and the expected benefits.

The purpose of this visit is to:

- to help him understand in which register this search for the analgesic function by vibrating massage is situated (the analgesic function is not the erotic function or the sexual function)
- To differentiate between the stimulation of the clitoris button for sexual purposes towards orgasm and the proposed procedure.
- inform the patient about the anatomical data acquired (diagrams taken from the 3D anatomy video of the female perineum, University of Lyon [21])
- describe the procedure (read Article 5.1 above).
- To enable them to better understand and frame the proposed practice in relation to their physical, personal and conjugal sexual practices.
- understand the risks (read paragraph 5.2 in full)

This exchange of information is essential to avoid misinterpretations, amalgams, confusions, shortcuts that never fail to pollute any discourse or care project as soon as the word "clitoris" appears in a conversation. This "dear stranger" from the film the same name [25] should be a little less so at the end of this visit in order to clarify the patient's consent, especially if the patient has never experienced an orgasm.

The proper use of the device is explained to the patient, as well as the risks associated with misuse.

Silicone allergy is tested (patient statement). In the event of an allergy, acceptability can be assessed but the device will not be given to the patient and she will therefore not be involved in the following steps.

The patient formulates all her questions.

It is the investigator's responsibility to verify the inclusion criteria for the study. It collects the patient's free consent and ensures that this consent is informed by the information already provided to V0.

It specifies the conditions of the massage episodes and the continuation of the protocol with regard to the limits that the patient wishes to set to the possible discovery of her own intimacy. To establish a climate of trust and serenity, he or she defines with her the confidentiality of the setting in which she wishes to participate in the study during particular episodes where this intimacy may be limited (in the hospital). For the time of childbirth work, he reassures the patient about the confidentiality of the place and in relation to the healthcare team (not always aware of the study).

For the rest of the delivery (expulsion and delivery) and only in the event that the patient chooses to continue with the protocol, he confirms the very necessary presence of one or both of the project investigators.

If the patient consents to participate, the patient and the investigator enter their first and last names, date and sign the consent form, a copy of which is given to the patient and the original kept by the investigator.
A telephone number and a dedicated email address are provided to the patient for any information during the duration of the study. A phone number will be requested to verify the proper functioning and use of the OVD provision through a telephone interview at the beginning of the last trimester of pregnancy.

the OVD (One Vibrating Device) is given to the patient.

The procedure is anatomically specified. The conditions of use (hygiene, duration) are explained by reading the leaflet (appendix 3) given with the object. The use of the OVD is again explained with a diagram given to the patient, taken from the video on the female perineum for the exact positioning (Appendix 1).

The 2 phone calls as well as the V2 visit are optional. If possible and depending on the term of the pregnancy, a date is set for telephone interviews.
The FAC schedule is given and explained to the patient.

6.6. Aftercare by phone call (2 Weeks after V1 if before childbirth if not NA)

The patient is contacted by the sexologist to verify the proper functioning of the OVD and to ensure its proper use.

6.7. Follow-up by phone call (beginning of the 3rd trimester if below 37 SA otherwise NA)

The patient is contacted by the sexologist to verify the proper functioning of the OVD and to ensure its proper use.

6.8. Follow-up visit – V2 (If patient included beyond 32 weeks, NA visit)

This V2 visit is scheduled for the 32nd week of amenorrhea in order to verify the understanding of the completion of the CAF calendar and answer any questions, in particular on the confidentiality conditions of the research.

6.9. End of Study Visit (last visit performed by the patient) – V3 This visit is scheduled postpartum, in the weeks following delivery for recovery of the CAF Calendar.

6.10. Rules for Stopping the Study

- *Premature, permanent or temporary termination of the entire Study*

The Study may be suspended or terminated by the sponsor or at the request of the Committee for the Protection of Persons (CPP) at any time.

- *For the included patient, premature, permanent or temporary termination of the Study*

Premature termination of the patient's participation in the study may be decided for the following reasons:

- in the event of termination of pregnancy, whether voluntary or involuntary,
- in the event of an unforeseeable or adverse event contraindicating the continuation of the study
- by decision of the investigator in the interest of the patient,
- in the case of a patient who is lost to follow-up,
- in the event of the patient's death.

Any premature termination will be dated and the reason for it will be reported by the investigator. The patient will no longer have a visit as part of the study with the exception of follow-up visits for possible adverse events.
In the event of an adverse event that warranted premature termination of the study, additional follow-up visits, appropriate to the event, will be conducted. Information on the course of the serious adverse event will be provided for the study.

- *By the included patient, premature, permanent or temporary termination of the study*

Since participation in the study does not imply a "mandatory" use of the tool, nor an "imposed" protocol during "imperative" massage sessions, the mere non-compliance with the program to investigate the analgesic function of the clitoris by itself is not a reason for discontinuation. This non-compliance is only noted on the CAF calendar and recorded as such.

However, any patient who wishes to formally withdraw from the study, for whatever reason, may withdraw her consent at any time and inform the investigator, who must make every effort to:

- Document the patient's decision in writing,
- Identify the reason(s) for leaving the study and report them in the CRF.

This withdrawal does not affect the use of data obtained on the basis of informed consent expressed before it was withdrawn.

7. Personal Safety

It is a study mentioned in 2° of Article L.1121-1 of the Public Health Code with minimal risks and constraints and the applicable reference is the health vigilance system (Article L.1123-10 of the Public Health Code).

The following expected potential risks to the individual are as follows:

- allergic reaction to silicone of One Vibrating Device (OVD)
- intrarectal or intravaginal foreign body in case of misuse of the OVD

<u>Management of Undesirable Events</u>

The investigator collects adverse events in the adverse event collection form located in the electronic CRF:

- The investigator evaluates each adverse event for its severity.
- The investigator evaluates the intensity of adverse events observed in the research participant and reports it in the case report, either using an adverse event gradation scale appended to the protocol (e.g. NCI-CTCAE version 5.0 (November 2017) or Clavien-Dindo for cancer trials), or by more general terms such as:

Lightweight (Grade 1)	Does not interfere with normal daily activity
Moderate (Grade 2)	partially restricts usual daily activity
Severe (Grade 3)	Limits usual daily activity
Very severe (grade 4)	life-threatening
Death (Grade 5)	

- The investigator evaluates the causal relationship of adverse events with the device being investigated

8. Statistics:

8.1. Description of Statistical Methods

The main analysis will be done by estimating the percentage of women who have undergone the procedure at least twice among those who have had the information visit.
From a statistical point of view, it will not be possible to keep detailed information on the women who came to the information visit but were not included; On the other hand, the volume of information visits can be accurately estimated by recording the number of visits.

A percentage above 25% will be considered acceptable.

The proportion of subjects who performed the procedure at least once at any time, among all women included will be assessed on an intention-to-treat basis. Thus, a topic with an incomplete timeline will be analyzed based on the available data. In the absence of data, it will be considered that the procedure has not been applied.

A safety analysis of adverse events will be carried out by simply describing the adverse effects and their number. Groupings of events will be carried out if necessary, in order to present a summary picture.

8.2. Expected number of people to be included in the Study

Since this is a feasibility study, the only data in the literature being very isolated reported cases, it is preferable to conduct the study on a small pilot sample. With 64 patients coming to the information visit, 32 included and 16 using the procedure, the uncertainty on the 16/64 rate will correspond to a factor of 2.5 between the upper and lower bound of the confidence interval.
That is, the expected confidence interval is (15% to 37%).

8.3. Expected Materiality

The primary analysis will be the estimation of a two-sided 95% confidence interval of a percentage. There is no degree of significance.

8.4. Statistical Criteria for Study Discontinuation

The study will be discontinued when the 32 patients scheduled for inclusion have reached their final V3 visit, unless there are difficulties in inclusion that prematurely terminate the study.

8.5. Replacement Arrangements

No replacements are planned.

8.6. Performing an interim analysis

No interim efficacy or safety analysis is planned. In the event of an adverse event, however, the study may be terminated prematurely, if patient safety is compromised.

9. Right of access to source data and documents.

The sponsor is responsible for obtaining the agreement of all parties involved in the study to ensure direct access to all study locations, source data, source documents and reports for quality control and audit purposes by the sponsor.

The investigators will put the documents and individual data strictly necessary for the follow-up, quality control and audit of research involving the person

available to persons having access to these documents in accordance with the regulatory and legislative provisions in force.

Source data is defined as any original document that can prove the existence or accuracy of data recorded during the test. In accordance with the legislative provisions in force (Articles L.1121-3 and R.5121-13 of the Public Health Code), persons with direct access to the source data will take all necessary precautions to ensure the confidentiality of information relating to the equipment used, the study, the persons who are suitable for it and in particular with regard to their identity and the results obtained. These persons, as well as the investigators themselves, are subject to professional secrecy.

During or after the human study, data collected on suitable individuals and provided to the sponsor by the investigators (or other specialized stakeholders) will be anonymized. Under no circumstances should they display the names of the persons concerned or their addresses in plain text. Only the healthcare professional who directs the conduct of the study can maintain the link between the coded identity of the persons taking part in the research and their first and last names.

The anonymized patient identification code will consist of a chronological inclusion number associated with the first letter of the last name and the first letter of the first name.
The sponsor will ensure that each person who participates in the study has been informed of their rights and how to exercise them. Likewise, it will ensure that each person taking part in the study has given his or her written consent to access the individual data concerning him or her that is strictly necessary for the quality control of the study.

As part of the Study, a computer processing of certain personal data of healthcare professionals, in accordance with MR001, will also be implemented to allow the implementation and conduct of the Study. For this purpose, this personal data will be transmitted to the Promoter or to persons or companies acting on its behalf, in France. This data may also, under conditions ensuring its confidentiality, be transmitted to French or foreign health authorities and to other entities of the sponsor.

This study is based on the reference methodology MR-001, relating to the management and transmission of the data collected during the study. The sponsor of this study has received a declaration of compliance with this reference methodology from the CNIL.

In accordance with the provisions of the Data Protection Act, Files and Civil Liberties, healthcare professionals have the right to access and rectify their computerized data at any time. They also have the right to object to the transmission of data covered by professional secrecy that may be used in the context of this study and processed.

10. Quality Control & Assurance

10.1. Study Data Management

10.1.1. Data entry and storage

When collected, the information concerning the patients participating in this study will be anonymized according to the recommendations established by the CNIL.

Clinical data will be entered into the secure online database developed by Rouen University Hospital using the Clinsight® software (Ennov Group, 251 rue du Faubourg Saint Martin in Paris 75010 - France). The data will be hosted on a secure server located at the Information System Department of Rouen University Hospital. Backup copies of the contents of this server are made on a daily basis.

The data will be entered by the investigating physician or the Clinical Study Technician, who will have personal and secure accounts (username and personalized password of at least 6 characters) for access to the study database. The persons performing the computer processing (biostatistician) will have access to the data once the database is frozen by making it available on a secure server accessible only to the data manager and the biostatistician of the study. Data entry will be carried out, under the responsibility of the investigator, by a member of his team (TEC, nurse,...).

10.1.2. Data processing, verification and validation

Once the data entry has been completed, terminal checks will be programmed according to the consistency tests programmed by the data manager. Data validation will be performed for statistical analysis, and requests for correction will be issued to the investigator or study TEC, who commits to complete and correct the data accordingly.

10.1.3. Recipient of the processed data

<u>The following categories of persons have access to the processed data:</u>

- The data controller and the persons acting on its behalf;

- The scientific manager (coordinating investigator) of the study;
- Healthcare professionals involved in the research and the staff acting under their responsibility (investigator, IRC, TEC of the investigator center, etc.);
- Persons responsible for quality assurance, i.e. responsible for monitoring and evaluating the quality and authenticity of the data collected;
- Legally authorized staff of health authorities and public control authorities, in the context of a particular mission or the exercise of a right of communication (ANSM, FDA, CNIL, etc.);
- Persons in charge of statistical analyses;
- Persons responsible for regulatory affairs and registration of research with competent authorities
- Authorized personnel acting under the responsibility of the insurance body guaranteeing the civil liability of the promoter.

All of these categories of persons are subject to professional secrecy.
The data may be passed on to contractual partners in a form that must not allow the direct or indirect identification of the persons involved in the research. The presentation of the results of the data processing may in no way allow the direct or indirect identification of the persons taking part in the study.

The sponsor, as data controller, implements procedures to ensure that all requests (access, rectification, opposition) from persons taking part in the study are complied with.

10.2. Quality control

The Clinical Research Associate, mandated by the sponsor, will make regular visits to the study center according to the monitoring plan established at the beginning of the study when:

- the implementation of the study,
- in the course of the study according to the rhythm of inclusions,
- at the end of the study.

The Clinical Research Associate will be responsible for ensuring that the rights and safety of the subjects are respected, the reliability, quality and traceability of the data and information transmitted, and to verify that the study is conducted in compliance with the protocol, GCP and the regulatory and legislative framework in force.
The purpose of the visits will therefore be to verify and validate:

- the data from the observation reports defined in the research monitoring plan
- Eligibility of included patients: compliance with inclusion and non-inclusion criteria
- the respect of patients information procedures and collection from their consents
- adherence to protocol-specific procedures, CAF schedule, LPC list, patient follow-up
- the quality of the data collected in the Case Report Form (CRF): accurate, complete and consistent
- compliance with SAE reporting procedures and developments
- the proper management and traceability of the study devices (accounting of OVD devices).

At the end of each visit, a standardized monitoring report will be drawn up by the ARC, which will be reviewed by the promoter.

10.3. Audit & Inspection

An audit can be carried out at any time by persons appointed by the sponsor and independent of the study leaders. Its objective is to ensure the quality of the study, the validity of its results and compliance with the law and regulations in force. The investigators agree to comply with the requirements of the sponsor and the Competent Authority with respect to an audit or inspection of the trial.

The audit can be applied to all stages of the study, from the development of the protocol to the publication of the results and the classification of the data used or produced in the study.

11. Retention and archiving of data relating to the Study

The sponsor and investigators archive essential documents and study-specific data in accordance with Good Clinical Practice **for a period of 25 years** following the end of the study.

The essential documents to be archived are:

- The Protocol and any amendments to the Protocol
- Case Reports
- Source records of participants who signed consent (only for the investigator)
- All other essential documents and correspondence related to the research, including the investigator binder.

The retention and archiving of all these respective documents is the responsibility of the investigator and the sponsor for the regulatory archival period. No removal or destruction may be carried out without the consent of the promoter. At the end of the statutory archiving period, the proponent will be consulted for destruction. All data, documents and reports are subject to audit or inspection.

12. Ethical and Regulatory Considerations

The sponsor, the investigator and the co-investigator undertake to ensure that this study is carried out in accordance with Law No. 2012-300 of 5 March 2012 on research involving human beings and its consolidated versions, as well as in accordance with Law No. 2004-806 of 9 August 2004 and its consolidated versions, Good Clinical Practices (I.C.H. in its consolidated version and decision of 24 November 2006) and the Declaration of Helsinki version of October 2008 (which can be found in its full version on the website http://www.wma.net).

The study is conducted in accordance with this protocol. Except in emergency situations requiring the implementation of specific therapeutic procedures, the investigator and the co-investigator undertake to comply with the protocol in all respects, in particular with regard to the collection of consent and the notification and follow-up of adverse events. This study received the favourable opinion of the Committee for the Protection of Persons (CPP), as *the name of the CPP*.

The Rouen University Hospital, the sponsor of this study, has taken out a civil liability insurance contract with Biomedic Insure in accordance with the provisions of Article L1121-10 of the Public Health Code.

The data recorded during this study are subject to computerized processing at Rouen University Hospital in compliance with Law No. 78-17 of 6 January 1978 relating to data processing, files and freedoms amended by Law 2004-801 of 6 August 2004 and its consolidated versions.

This study falls within the framework of the "Reference Methodology" (MR-001) in application of the provisions of Article 54 paragraph 5 of the amended Law of 6 January 1978 relating to information, files and freedoms. This change was approved by decision of 5 January 2006. The Rouen University Hospital has signed a commitment to the CNIL to comply with this "Reference Methodology".
This study is registered on the website http://clinicaltrials.gov/

Amendment to the Protocol: An amendment is a substantial change.

Any substantial modification, i.e. any modification likely to have a significant impact on the protection of individuals, on the conditions of validity and on the results of the research, on the quality and safety of the tools tested, on the interpretation of the scientific documents that support the conduct of the research or on the methods of conducting it, is subject to a written amendment that is submitted to the proponent; Depending *on the type of modification, the user must:*

- Either obtain, prior to its implementation, a favourable opinion from the PPC,
- *Or be forwarded to the PPC for information*

All amendments to the protocol must be brought to the attention of the investigator and the co-investigator, who undertake to respect the content by signing a membership document in the event of a modification of the protocol.

Any amendment that modifies the management of patients or the benefits, risks and constraints of research is the subject of a new information note and a new consent form, the collection of which follows the same procedure as the one mentioned above.

13. Rules on publication and communication.

The analysis of the results will be the subject of conference papers and publications. The text of publications and communications will be discussed with all investigators participating in the trial. The co-authors are the principal investigator and co-investigator, Professor Eric VERSPYCK and Dr. Marie-Madeleine, Manon BESTAUX-BRETHEZ. The publishing rules are as follows:

In French:

Centre Hospitalier Universitaire (CHU) de Rouen, Department of Gynecology-Obstetrics, Rouen, F 76000, France.

Centre Hospitalier Universitaire (CHU) de Rouen, Biostatistics Unit, Rouen, F 76000, France.

In English:

Rouen University Hospital, department of Obstetrics & Gynaecology, Rouen, F 76000, France.

Rouen University Hospital, department of Biostatistics, Rouen, F 76000, France.

14. References to scientific literature

1. Schick V., Herbenick D, Jozkowski KN, Jawed-Wessel S, Reece M. The sexual consumer: characteristics, expectations, ans experinences of women attending in - home sex toy parties. J Sex Marital Ther. 2013; 39(2) : 160-75.
2. Meston CM, Frohlich PF. The Neurobiology of Sexual Function. Arch Gen Psychiatry. 2000 Nov 1; 57(11):1012–30.
3. O'Connell HE, Hutson JM, Anderson CR, Plenter RJ. Anatomical relationship between urethra and clitoris. J Urol. 1998 Jun; 159(6):1892–7.
4. Foldes P, Buisson O. The clitoral complex: a dynamic sonographic study. J Sex Med. 2009 May; 6(5):1223–31.
5. Whipple B, Komisaruk BR. Analgesia produced in women by genital self-stimulation. J Sex Res. 1988 Jan; 24(1):130–40.
6. Vuillet Anna. Does masturbation have an analgesic role to play in childbirth? (Work and Expulsion Phases) [Master's thesis for the Bachelor's degree in midwifery]. [Angleur (BE)]: Haute Ecole Libre Mosane Sainte-Julienne; 2018.
7. Carmichael MS, Humbert R, Dixen J, Palmisano G, Greenleaf W, Davidson JM. Plasma oxytocin increases in the human sexual response. J Clin Endocrinol Metab. 1987 Jan; 64(1):27-31.
8. Thackare H1, Nicholson HD, Whittington K. Oxytocin--its role in male reproduction and new potential therapeutic uses. 2006 Jul-Aug; 12(4):437-48. Epub 2006 Jan 25.
9. Caruso S1,2, Mauro D1,2, Scalia G3, Palermo CI3, Rapisarda AMC1,2, Cianci A1,2 Oxytocin plasma levels in orgasmic and anorgasmic women Gynecol Endocrinol. 2018 Jan; 34(1):69-72. doi: 10.1080/09513590.2017.1336219. Epub 2017 Jun 11
10. Skrundz M[1], Bolten M, Nast I, Hellhammer DH, Meinlschmidt G. Plasma oxytocin concentration during pregnancy is associated with development of postpartum depression. Neuropsychopharmacology.2011Aug; 36(9):1886-93. doi:10.1038/npp.2011.74. Epub 2011 May 11.
11. Isabelle Lacroix Ph.D. Thesis in Pharmacology Pharmacovigilance in Pregnant Women: Maternal and Neonatal Aspects (Example of Psychoactive Substances) Toulouse 2009 http://thesesups.ups-tlse.fr/579/1/Lacroix_Isabelle.pdf
12. Claire Gricourt. The work led by oxytocin synthesis at the University Hospital of Rouen. Retrospective and single-center study. About 169 cases. Gynecology and obstetrics. 2011. DUMAS-00631182 https://dumas.ccsd.cnrs.fr/dumas-00631182
13. Tyzio R[1], Nardou R, Ferrari DC, Tsintsadze T, Shahrokhi A, Eftekhari S, Khalilov I, Tsintsadze V, Brouchoud C, Chazal G, Lemonnier E, Lozovaya N, Burnashev N, Ben-Ari Y Oxytocin-mediated GABA inhibition during delivery attenuates autism

pathogenesis in rodent offspring. Science. 2014 Feb 7; 343(6171):675-9. DOI: 10.1126/science.1247190.

14. Peng Tan; Choon Yow; Siti Omar. doi.org/10.1097/01.AOG.0000267201.70965.ec Effect of Coital Activity on Onset of Labor in Women Scheduled for Labor Induction: A Randomized Controlled Trial Obstetrics & Gynecology. 110(4):820-826, OCT 2007
15. NS Omar , PC Tan , N Sabir , ES Yusop , SZ Omar Coitus to expedite the onset of labour: a randomised trial General obstetrics 2012 https://doi.org/10.1111/1471-0528.12054
16. Peng Tan; Anggeriana Andi; Noor Azmi; M Noraihan Effect of Coitus at Term on Length of Gestation, Induction of Labor, and Mode of Delivery Obstetrics & Gynecology. 108(1):134-140, JULY 2006 DOI: 10.1097/01.AOG.0000223229.83920.af
17. Bajos N, Bozon M, Beltzer N. Survey on sexuality in France: practices, gender and health. Editions La Découverte; 2008.
18. Ronald Melzack and Patrick D. Walles, The gate control theory of pain, 1965. https://doi.org/10.1016/S1082-3174(96)80050-X .

19 Roll Jean-Pierre, Transcutaneous Vibrational Stimulation and Pain, 2019 https://doi.org/10.1016/j.douler.2019.09.004

20. Hegde KM, R N, Srinivasan I, D R MK, Melwani A, Radhakrishna S, Effect of vibration during local anesthesia administration on pain, anxiety, and behavior of pediatric patients aged 6-11 years: A crossover split-mouth study, J Dent Anesth Pain Med. 2019 Jun; 19(3):143-149. doi: 10.17245/jdapm.2019.19.3.143. Epub 2019 Jun 30
21. Patrice THIRIET Institute of Rehabilitation Sciences and Techniques - University of Lyon 1 video of 3D anatomy of the female perineum University of Lyon, 2015 https://www.youtube.com/watch?v=9psQO8RNfXw
22. William.H. Masters and Virginia. E. Johnson, *Human Sexual Response*, Little, Brown and Co, 1966 (ISBN 0-3165-4987-8).
23. Postel T. Birth and Orgasm: Evidence of the Existence of an Obstetric Orgasm. Sexologies. 2013 Oct 1; 22(4):165–8.
24. Bonnet S, Caltero C, Dessogne F, Fanchette M-P, Fawer K, Ferchichi R, et al. Birth of an analogue visual scale. 14th Congress of the SFETD; 2014 Nov 22; Toulouse.
25. Film "The Clitoris That Dear Stranger" 2003 - Michèle Dominici, Variety Moszinski and Stephen Firmin Hum Reprod Update.

15. List of abbreviations

SA: Amenorrhea Week

CAF: Clitoris Analgesic Function

ACC: Childbirth

OVD : One Vibrating Device

CE: Compliance with European standards

PP: Postpartum

CALL: Telephone interview at the beginning of the 3rd trimester

16. List of Appendices:

- Appendix 1 = photo of OVD positioning, according to photo taken from the video of the University of Lyon on the female perineum in 3D [16],

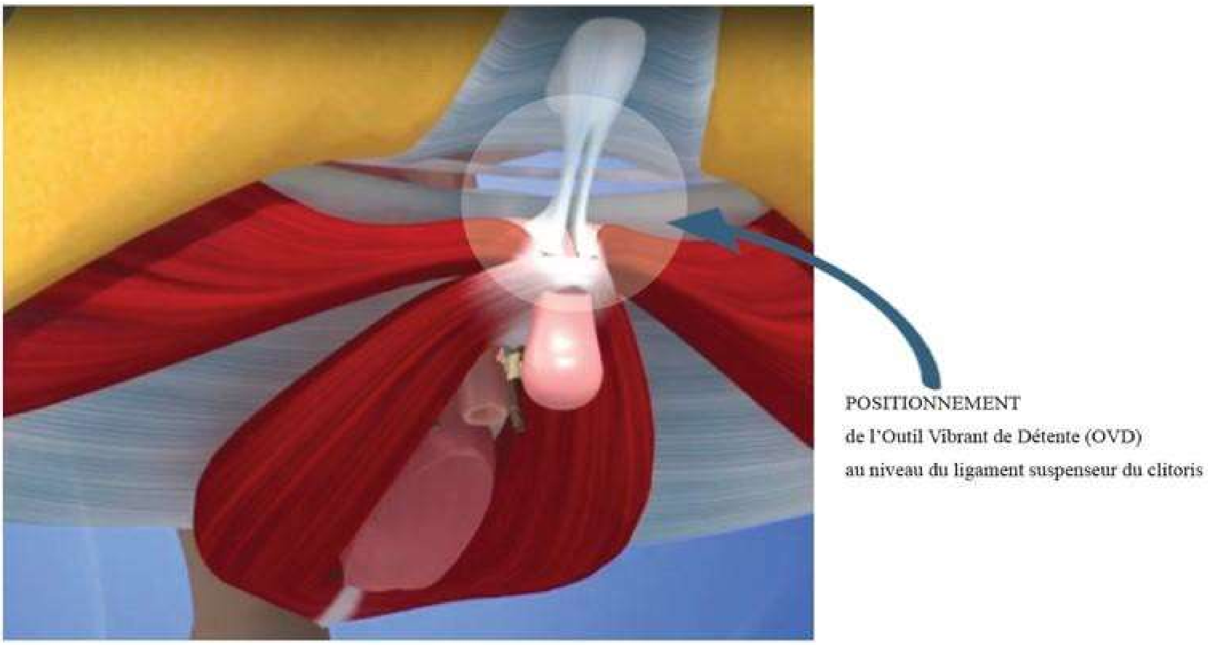

- Appendix 2 = document given to all pregnant patients at the first pregnancy consultation,

- PAIN MANAGEMENT -
Would you like to participate in medical research?

Rouen University Hospital is currently conducting a medical research project on the relief of obstetric pain during pregnancy and childbirth.
by a non-drug method.

If you would like further information before deciding to participate, please make an appointment at the secretariat of the gynaecology and obstetrics department for an information consultation with Dr Manon BESTAUX,
Pavillon Martainville, 02 32 88 84 22

You can bring a person of your choice with you.

- Appendix 3 = OVD (Vibrating Trigger Tool) CE marked.

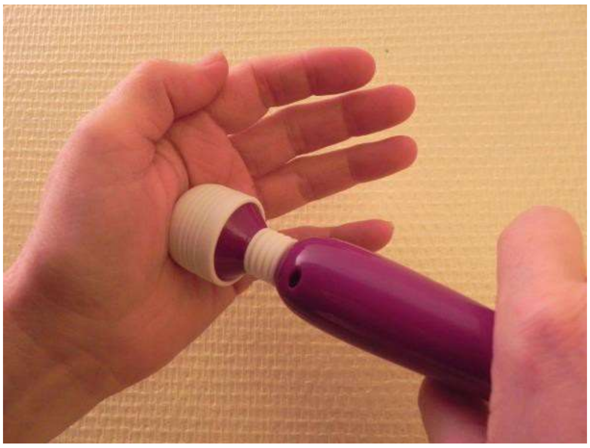

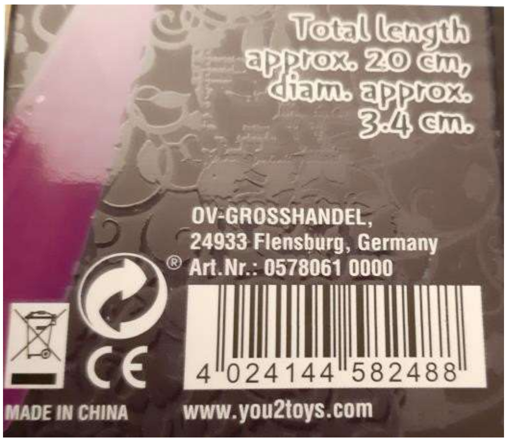

Notice d'utilisation et d'entretien du lovetoy vibrant

1. Nettoyez le lovetoy vibrant à l'eau chaude et au savon avant la première et après chaque nouvelle utilisation. Après chaque utilisation anale, l'appareil doit être nettoyé en profondeur avant de pouvoir être utilisé au niveau vaginal.
   Soyez prudents – n'oubliez pas qu'il s'agit d'un appareil électrique, l'humidité dans le compartiment à piles peut causer des dommages.
2. Appliquez assez de lubrifiant sur la surface du lovetoy vibrant. Veillez en ce faisant à ce que l'unité de contrôle fonctionnant avec des piles ne soit pas endommagée, car cela pourrait entraîner un mauvais fonctionnement de l'appareil. ***IMPORTANT :*** n'utilisez que du lubrifiant soluble à l'eau ! Les lubrifiants à base d'huile attaquent les matériaux.
3. Après l'avoir posé ou introduit en douceur, appuyez ou tournez avec précaution le contrôle de vitesse et sélectionnez la vitesse de vibration souhaitée ou choisissez une fonction de l'unité de contrôle qui correspond à vos souhaits. Tournez, appuyez ou poussez le régulateur de vitesse lentement et en douceur, jamais violemment.
4. Eteignez le lovetoy vibrant après utilisation et retirez-le doucement de votre corps.
5. Avant de le ranger, laissez refroidir le lovetoy vibrant après utilisation.
6. Gardez le lovetoy vibrant au propre, au frais et au sec, car l'humidité peut endommager les piles et le moteur. Protéger des rayons du soleil !
7. Rangez votre lovetoy vibrant séparément et pas avec des articles composés de matières différentes afin d'éviter des réactions chimiques.

Avertissements et remarques générales :

1. Conserver hors de la portée des enfants. Les petites pièces peuvent être avalées.
2. Ne pas utiliser le lovetoy vibrant en cas de maladie ou d'irritation de la peau ou sur une peau infectée.
3. Afin d'éviter les maladies et les infections, ne transmettez pas le lovetoy vibrant à un tiers.
4. Veillez respecter les notes relatives aux matériaux indiquées sur l'emballage afin d'éviter les réactions allergiques, comme par ex. au latex entre autres.
5. Assurez-vous que les piles sont bien placées correctement dans le compartiment à piles. Vérifiez les polarités + et – et placez les piles dans le bon sens.
6. Lorsque le lovetoy vibrant est imperméable, vérifiez que le compartiment est fermé hermétiquement après avoir placé les piles.
7. En cas de surchauffe du lovetoy vibrant, éteignez-le immédiatement et laissez-le refroidir avant de l'utiliser à nouveau.
8. N'essayez pas de recharger des piles non rechargeables.
9. Ne jetez pas les piles dans le feu.
10. Ne pliez, ni ne détendez pas trop le Lovetoy. N'employez pas la force.
11. N'utilisez le lovetoy vibrant que pour accompagner des préliminaires partagés, comme appareil de massage ou de stimulation, pour la masturbation ou pour accompagner les rapports sexuels et ne l'utilisez pas dans d'autres buts.
12. Le fournisseur n'assume aucune responsabilité pour l'article, si celui-ci n'est pas utilisé correctement.
13. Le produit n'est pas destiné à un usage professionnel.

Note concernant la protection de l'environnement :

ne pas jeter les emballages, ni les appareils usagés, mais les amener aux points de collecte. Veuillez remettre les appareils usagés conformément à votre législation locale et aux directives 2002/96/EG et 2006/66/EG aux points de collectes respectifs.
Le sigle de la poubelle à roulette barrée (🗑) signifie que cet appareil doit être remis à un centre de collecte pour appareils électroménagers usagés afin d'assurer le meilleur recyclage des matières premières.

Retour des piles

La place des piles n'est pas dans la poubelle de la maison. En tant qu'utilisateur, vous êtes légalement tenus de ramener les piles usagées, par exemple dans les déchetteries publiques ou là où les piles vous ont été vendues. Les piles des catégories mentionnées ici peuvent nous être renvoyées gratuitement. Les piles contenant des substances toxiques portent le signe 🗑 et l'un des symboles chimiques Cd (la pile contient du cadmium), Hg (la pile contient du mercure) ou Pb (la pile contient du plomb). Les piles au lithium et les piles rechargeables de tous les systèmes ne doivent être rendues au centre de collecte que déchargées. La polarité de ce type de piles doit être recouverte de bande adhésive avant d'être remises au centre de collecte pour éviter les courts-circuits provoqués en cas de non déchargement total des piles.

- Appendix 4 = CAF Calendar

Nom :
Prénom :

Mois :

Protocole FAC - CHU de Rouen

Jour du mois	Moment de douleur				Contraction			Intensité de la douleur * *(entre 1 et 10)*	Utilisation procédure		SI UTILISATION PROCEDURE			Impressions ou Commentaires *(facultatif)*
	Matin	Après Midi	Soir	Nuit	Oui	Non	Ne sait pas		Oui	Non	soulagement Oui	soulagement Non	Intensité de la douleur *	
Ex : 10	X						X	7	X		X		2	*heureusement j'étais chez moi*

intensité de la douleur :

Pas de douleur

Douleur maximum imaginable

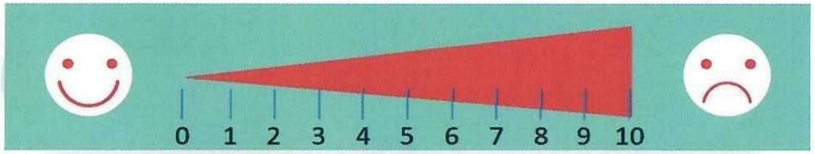

Plus le trait est proche de cette extrêmité, **MOINS** la douleur est **FORTE**.

Plus le trait est proche de cette extrêmité, **PLUS** la douleur est **FORTE**.
